# Selective aerobic oxidation of alcohols with supported Pt nanoparticles: effect of particle size and bismuth promotion

**DOI:** 10.1039/d5sc07190a

**Published:** 2025-10-28

**Authors:** Anna Giorgia Nobile, Enzo Brack, Milivoj Plodinec, Christophe Copéret

**Affiliations:** a Department of Chemistry and Applied Biosciences, ETH Zurich Vladimir-Prelog-Weg 2 8093 Zurich Switzerland ccoperet@ethz.ch; b Scientific Center for Optical and Electron Microscopy, ETH Zurich Otto-Stern-Weg 3 CH-8093 Zurich Switzerland

## Abstract

Oxidation reactions are a cornerstone of the chemical industry, enabling the synthesis of bulk commodities and high-value fine chemicals, where catalytic processes play a crucial role. With the demand to establish more sustainable approaches, developing more selective and robust aerobic oxidation processes, particularly using heterogeneous catalysis, remains of contemporary relevance. Herein, we report the synthesis of well-defined PtBi nanoparticles supported on functionalized carbon *via* surface organometallic chemistry (SOMC) and their use as heterogeneous catalysts for aerobic alcohol oxidation. Our study demonstrates that alloying of Pt with Bi, as well as increasing the nanoparticle size, enhances the catalytic stability and mitigates active-site poisoning during aerobic alcohol oxidation. The best-performing catalyst reaches 93% conversion and 98% selectivity over multiple cycles in the oxidation of prenol, an industrially relevant intermediate.

## Abstract 2

While small Pt(Bi) nanoparticles supported on carbon suffer from poisoning and large Pt nanoparticles suffer from sintering, large PtBi/C nanoparticles show high performance and stability for aerobic alcohol oxidation.

## Graphical abstract

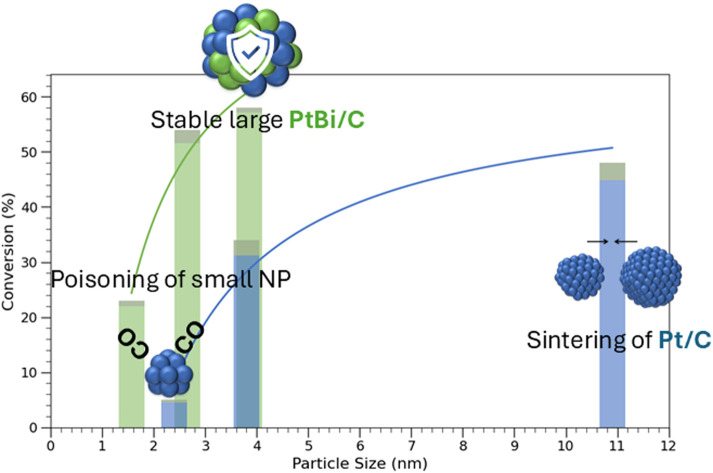

## Introduction

Oxidation reactions are among the most essential processes employed in the chemical industry, with applications ranging from the synthesis of bulk chemicals (*e.g.*, terephthalic acid, acrylate derivatives, propene oxide…) to fine chemicals (fragrance, vitamins, and pharmaceuticals). These processes also play a key role in the conversion of alternative feedstocks, *e.g.*, biomass, for the production of value-added chemicals.^1–10^ Over the years, selective oxidation has relied more and more on catalytic processes, and over time transitioned to greener oxidants, such as H_2_O_2_ and O_2_.^2^ Prototypical examples are the synthesis of propene oxide using TS1 in combination with H_2_O_2_ or the oxidation of *n*-butane to maleic anhydride using vanadyl phosphate and O_2_.^11–14^

**Fig. 1:**
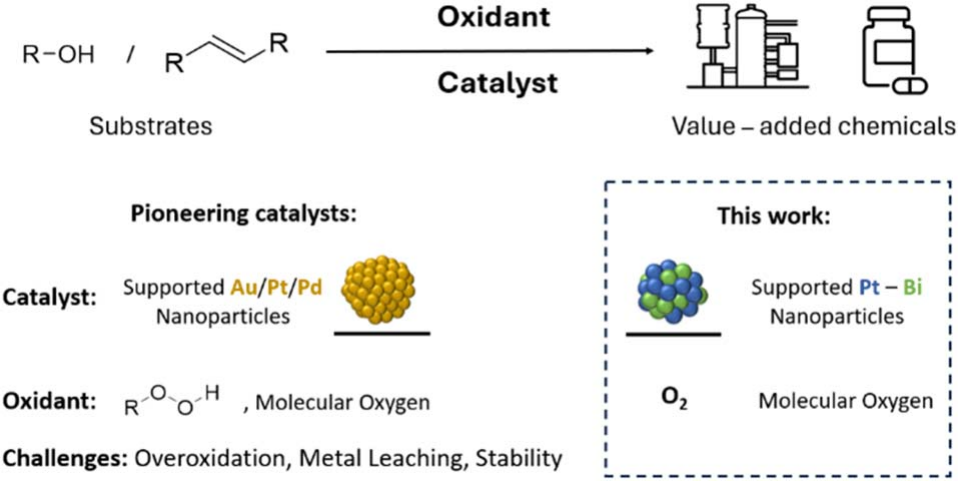
(Top) Oxidation of alcohols and olefins to produce value-added chemicals. (Bottom) Conventional catalysts rely on supported precious metals and peroxides or O_2_, but often suffer from overoxidation and metal leaching. Here, carbon-supported PtBi nanoparticles enable selective oxidation with O_2_ and improved catalytic stability.

The growing need for more sustainable processes has intensified interest in aerobic oxidation, since O_2_ is abundant, inexpensive, and ideally yields only water as a byproduct. While the use of O_2_ may also lead to safety concerns, the associated risks can be alleviated with appropriate measures. Despite its clear advantages, O_2_ exhibits low reactivity due to its triplet ground state.^2^ To increase its reactivity, homogeneous and heterogeneous catalysts are typically employed under thermal, photo-, or electro-activation conditions.^3^ In the same context, the transition from homogenous to heterogeneous oxidation catalysts has attracted a lot of interest for process intensification.^15–17^ As a consequence, significant research efforts have focused on the use of supported metal nanoparticles (NPs), which exhibit higher selectivity and stability compared to metal oxide catalysts.^18^ Prominent pioneering examples have highlighted the potential of Pd, Pt, and Au NPs as catalysts for the selective aerobic oxidation of alcohols (see Fig. 1).^19–25^ However, these monometallic systems often suffer from lower selectivity and stability due to overoxidation and metal leaching.^2,3^ To overcome these issues, dopants, also called promoters, are commonly introduced to improve catalyst performances, as shown for the bimetallic AuPd/TiO_2_.^26^ The use of more abundant elements has also been investigated for aerobic alcohol oxidation because of their low cost and higher sustainability.^19,27–29^ Among those, bismuth is particularly noteworthy because it improves the catalytic performances of carbon-supported Pt NPs across oxidation reactions.^4,30–32^

While electronic and steric properties of this heavy pnictogen (Bi) have been proposed to improve catalytic performances, the exact role of Bi-promotion remains debated. For instance, higher conversion and selectivity have been reported in the Bi-promoted oxidation of glycerol.^9,33,34^ Notably, Bi can be readily found in different oxidation states with reversible redox chemistry, Bi(*n*) ↔ Bi(*n* + 2);^35^ this may explain why Bi is effective at preventing overoxidation.^36–39^ Furthermore, geometric blocking induced by Bi might help prevent poisoning of Pt-surface sites by the product.^40^ Overall, the promotional role of Bi remains elusive due to structural complexity, which often originates from the preparation methods themselves, *e.g.*, impregnation or coprecipitation, during which dissolution and precipitation events lead to the incorporation of metal sites in various locations and states.

Herein, we reason that surface organometallic chemistry (SOMC) could alleviate some of these challenges and enable to generate tailored PtBi NPs supported on activated carbon (Fig. 1).^41^ Notably, the prepared carbon-supported PtBi/C catalysts show an enhanced performance in the aerobic oxidation of 2-methoxy ethanol, a prototypical substrate, with respect to their monometallic analogues. Noteworthy, we observe that larger NP size and the presence of Bi greatly improve catalytic performances of Pt-based catalysts; in particular, the stability towards Pt leaching and NPs sintering, due to alloying and controlled Pt oxidation state. We further showcase their use in the oxidation of prenol – an industrially relevant substrate – where the presence of Bi enables the preservation of catalytic performances over multiple cycles by suppressing sintering.

## Results and discussion

**Fig. 2:**
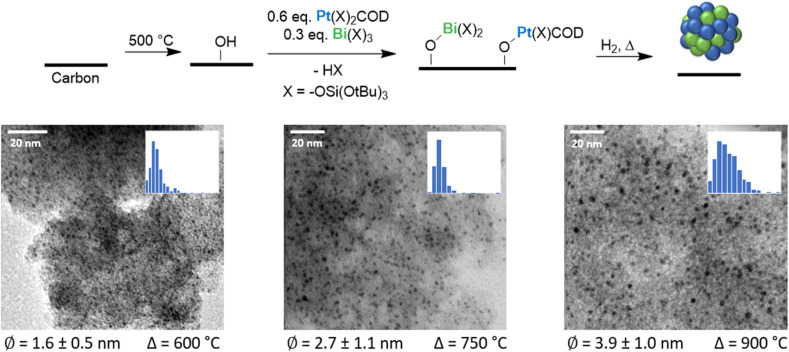
(Top) Schematic preparation of the PtBi/C catalysts *via* co-grafting. The carbon support is first calcined at 500 °C, resulting in surface reactive sites for the grafting of Pt and Bi precursors. Subsequent exposure to a flow of H_2_ at elevated temperatures leads to the formation of bimetallic PtBi nanoparticles. (Bottom) ABF STEM images and corresponding particle size distributions of PtBi/C prepared at different temperatures.

All catalysts are prepared using a mesoporous carbon support with high porosity and a specific surface area of 767 m^2^ g^−1^ (see SI 2.1), as well as a tunable surface chemistry. These characteristics enable a good dispersion of the metals over the support by preventing agglomeration of the formed NPs while minimizing mass-transport limitations.^42^ To functionalize the carbon support with surface oxygen-functional groups – primarily reactive OH groups – the carbon support is first treated in atmospheric air for 1 hour at 500 °C.^43^ Titration with [Mg(Bn)_2_(THF)_2_] shows the presence of 0.17 mmol g^−1^ reactive sites (0.13 sites per nm^2^). The presence of such sites facilitates the anchoring of molecular precursors *via* a SOMC approach while enabling control over the density and distribution of metal sites during NPs formation upon hydrogen treatment.^41^ Next, Bi and Pt are introduced by using [Bi(OSi(O^*t*^Bu)_3_)_3_] and [Pt(COD)(OSi(O^*t*^Bu)_3_)_2_] as thermolytic molecular precursors, since tris-(*tert*-butoxy)-siloxy (OSi(O^*t*^Bu)_3_) ligands are known to readily decompose under heat treatment at high temperatures, leaving silica as the sole co-product.^15,44^ For the preparation of the bimetallic PtBi/C catalysts, both metal precursors are co-grafted onto the carbon support – 0.7 equiv. of Pt and 0.3 equiv. of Bi per surface OH groups (see Fig. 2), aiming for a Pt : Bi ratio close to 3/1, which has been previously reported to best promote alcohol oxidation.^45,46^ Monometallic materials are prepared by grafting 1 equiv. of the respective precursors. Subsequent exposure of the materials to a flow of H_2_ at 1 bar and 600 °C yields supported monometallic Pt/C_600°C_ and Bi/C_600°C_, as well as bimetallic PtBi/C_600°C_ NPs (the catalysts are designated as M/C_*T*°C,_ and their preparation and characterization are provided in the SI 2 and 3 respectively), with 3.02 Pt wt%, 2.04 Bi wt%, and 3.42 Pt wt% with 1.30 Bi wt% metal loadings, respectively. The monometallic Pt/C_600°C_ (3.02 Pt wt%) displays NPs with a size of 2.4 ± 0.7 nm, while the bimetallic PtBi/C_600°C_ counterpart shows smaller NPs exhibiting a size of 1.6 ± 0.5 nm as visible from annular bright-field scanning transmission electron microscopy (ABF-STEM) images (Fig. 2). In contrast, the monometallic Bi/C_600°C_ displays very large particles (0.05–20 μm) with a highly heterogeneous size distribution (see Fig. S7 and 8), highlighting the synergistic effect between Bi and Pt in controlling the NPs size in bimetallic PtBi systems. Moreover, to investigate the effect of the particle size on the alcohol oxidation performance – a descriptor of interest for this reaction – Pt/C and PtBi/C catalysts with larger mean particle size are also prepared by varying the temperature during the hydrogen treatment from 600 °C to 750 °C and 900 °C. Hydrogen treatment at higher temperatures results in the formation of larger NPs, as observed from ABF-STEM images (see Fig. 2). Monometallic NPs exhibit a significant increase in mean particle size from 2.4 ± 0.7 nm (Pt/C_600°C_, Fig. S2) to 10.9 ± 9.8 nm (Pt/C_900°C_, Fig. S5). More surprisingly, Pt/C_900°C_ NPs exhibit an unusually large lattice spacing (*d*-spacing) of 0.39 nm (Fig. S5), suggesting the formation of Pt carbide, paralleling what has been reported for Pt NPs during the growth of carbon nanotubes.^47^ While smaller Pt NPs have an FCC structure, the larger NPs cannot be matched to any known Pt, Pt–C, or Pt–Si structures. In sharp contrast, bimetallic PtBi/C_900°C_ NPs show a moderate size increase to 3.9 ± 1.0 nm, compared to PtBi/C_600°C_ (1.6 ± 0.5 nm) (see Fig. 2). The *d*-spacing of both small and larger PtBi NPs remains similar (up to 0.22 nm, Fig. S12), indicating no significant structural changes at high temperature. The reduced level of sintering observed in PtBi/C suggests a higher thermal stability of the bimetallic PtBi/C catalysts compared to their monometallic Pt/C counterpart.

Next, we characterize the prepared catalysts using H_2_ and CO chemisorption, STEM electron dispersive X-ray spectroscopy (STEM-EDX), and X-ray absorption spectroscopy (XAS) to gain insight into their composition, surface reactivity, alloying, and oxidation states. Note that chemisorption experiments are performed on H_2_-treated samples, while XAS measurements have been carried out on air-exposed catalysts, as prior to the catalytic tests (see SI for experimental details). At 600 °C, the bimetallic PtBi/C_600°C_ catalyst exhibits a significantly higher H_2_ chemisorption capacity (2.91 mmol H_2_ per g Pt) compared to monometallic Pt/C_600°C_ (0.15 mmol H_2_ per g Pt), indicating an increased number of surface Pt sites per gram of catalyst upon addition of Bi. This difference is most likely linked to the smaller NPs size of the bimetallic sample, as well as possible electronic effects (*vide infra*). Interestingly, despite Pt/C_900°C_ exhibiting much larger NPs, it adsorbs a similar amount of H_2_ (0.14 mmol H_2_ per g Pt) as Pt/C_600°C_, possibly due to structural changes of the NPs' morphology and presence of Pt carbides, as highlighted by the substantial variations in the *d*-spacing (*vide supra*). Notably, the bimetallic PtBi/C_900°C_ shows a substantial decrease in H_2_ uptake (0.26 mmol H_2_ per g Pt) relative to PtBi/C_600°C_ (2.91 mmol H_2_ per g Pt), consistent with the larger mean NPs size and reduced surface to bulk ratio (see Table S15). Interestingly, both materials treated at 900 °C show very similar H_2_ chemisorption capacity, despite their markedly different NPs sizes. Furthermore, STEM-EDX elemental analysis of the bimetallic PtBi/C catalysts treated at different temperatures shows a spatial overlap of the Pt M and Bi M signals, suggesting the formation of a Pt–Bi alloy upon H_2_ treatment (see SI 3.2). To further probe alloying, we next turn to CO chemisorption experiments. Despite PtBi/C_600°C_ exhibiting smaller NPs compared to Pt/C_600°C_, a lower CO uptake (0.98 *vs.* 1.52 mmol CO per g Pt) is observed, suggesting an intrinsic difference in the surface composition of the NPs (see Table S16). As IR studies are not readily feasible on carbon-supported materials due to their inherently high absorption, we also prepare the analogous silica-supported PtBi/SiO_2–600°C_ catalyst (see Fig. S14 and S17), which exhibits NPs with a comparable size to PtBi/C_750°C_ (2.9 nm *vs.* 2.7 nm). CO-IR adsorption studies on the silica-supported material (Fig. S18) show a redshift in the CO stretching frequency from 2084 cm^−1^ (Pt/SiO_2–600°C_)^48^ to 2028 cm^−1^ (PtBi/SiO_2–600°C_), indicating an electronic interaction between Pt and Bi, where Bi donates electron density to Pt (*vide infra*). These observations are in agreement with the previously discussed results obtained by STEM-EDX and further corroborate the formation of PtBi alloys during reduction. We then perform XAS measurements on the air-exposed catalysts at the Bi L_3_ and L_2_ edges, as well as Pt L_3_ edge, to gain further insight into differences between the catalysts prepared at 600 °C and 900 °C (note that the Bi L_3_ edge is influenced by the EXAFS region of the Pt L_2_ edge; see SI 3.5 for additional details). At the Pt L_3_ edge (see Fig. S19), all samples exhibit a white line intensity between that of Pt foil and [Pt(COD)(OSi(O^*t*^Bu)_3_)_2_], consistent with Pt oxidation states between 0 and +II.^44,48^ Catalysts treated at higher temperatures exhibit slightly lower white line intensities, indicating a more reduced state and suggesting a higher stability of Pt(Bi)/C_900°C_ towards Pt oxidation, when exposed to air. Comparing monometallic and bimetallic samples, the latter show a small shift to lower energy at the Pt L_3_ edge, indicating electron transfer from Bi to the Pt 5d band, supporting the formation of a PtBi alloy, paralleling what is observed for PtGa/C catalysts.^44^ Fourier-transformed EXAFS data (Fig. S19) reveal a prominent Pt–O feature at 1.60 Å for samples treated at 600 °C (Pt(Bi)/C_600°C_), consistent with Pt(II) sites, and in agreement with the observed higher white line intensity. High-temperature samples (Pt(Bi)/C_900°C_) display an additional feature at 2.23 Å, likely corresponding to the formation of Pt–C bonds.^49^ These findings further corroborate the presence of Pt carbides, consistent with the observation of an unusually large *d*-spacing of 0.39 nm (*vide supra*). Notably, no direct Pt–Pt coordination is observed in any sample, suggesting that O, C, and Bi are intercalated within the air-exposed Pt NPs. Analysis of the Bi L_2_ edge in the prepared bimetallic PtBi/C materials (Fig. S21), Bi_2_O_3,_ and NaBiO_3,_ reveals a slightly higher edge energy for PtBi/C_600°C_ compared to PtBi/C_900°C_, consistent with Bi remaining slightly more oxidized. Overall, these results highlight differences in the electronic structure of the catalysts prepared at different temperatures, as well as upon alloying of Pt and Bi. The incorporation of Bi results in higher thermal stability against NP growth, while the H_2_ treatment at higher temperatures shows a lower extent of oxidation of Pt and Bi, as well as the possible intercalation of C into the NPs' structure.

**Fig. 3:**
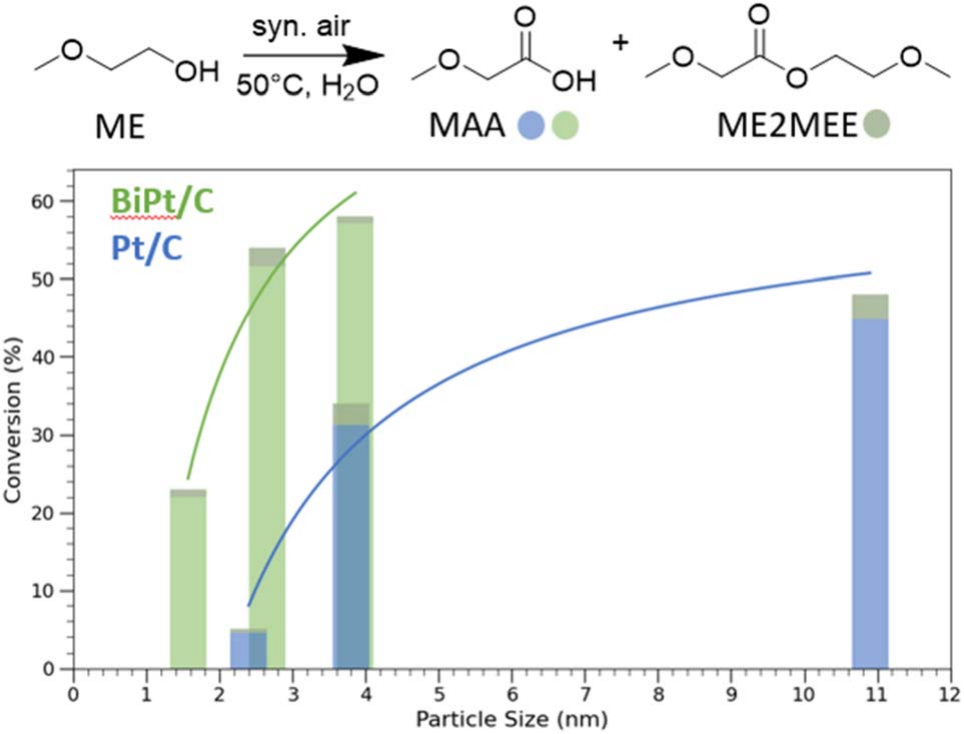
(Top) Schematic selective oxidation of 2-methoxy ethanol (ME) to 2-methoxacetic acid (MAA). (Bottom) Oxidation conversion of monometallic Pt/C (blue) and bimetallic PtBi/C (green) as a function of the NP size. The coloured part of the bar represents the conversion to MAA, while the grey part is the conversion to the corresponding ester (2-methoxyethyl)-2-methoxyacetate (ME2MEE).

Next, we evaluate the catalytic performances of these materials for the oxidation of 2-methoxyethanol under aqueous conditions using synthetic air as the oxidant. All reactions are conducted at 50 °C for 9 hours with 30 V% of 2-methoxyethanol in water and a catalyst loading corresponding to 0.2 mol% Pt relative to 2-methoxyethanol (see SI 4 for more details). Under aerobic conditions (5 bar synthetic air), all Pt-containing materials catalyze the conversion of 2-methoxyethanol to the corresponding carboxylic acid, while the Bi/C_600°C_ catalyst shows no activity. As seen in Fig. 3, the conversion rates for both monometallic Pt/C and bimetallic PtBi/C catalysts show a particle size dependence. Pt/C_600°C_ and PtBi/C_600°C_ exhibit a conversion of 5.2% and 22.8% respectively, while for Pt/C_900°C_ (48.4%) and PtBi/C_900°C_ (58.3%), a tenfold and twofold increase in conversions is observed. Thus, in both systems, larger NPs outperform significantly the smaller ones. Notably, a slight increase in conversion as well as higher selectivity (98.6% *vs.* 93.6%) is observed for PtBi/C_900°C_ with respect to Pt/C_900°C_ despite the much higher conversion of the former. These data correlate with the lower degree of oxidation of Pt and Bi observed by XAS for Pt(Bi)/C_900°C_, highlighting the deactivating effect of Pt(II) sites. Surprisingly, the high H_2_ uptake of PtBi/C_600°C_, which suggests a large number of accessible Pt surface sites, does not translate into high catalytic activity. As previously suggested, the presence of a too large proportion of surface Pt(0) sites might lead to active site poisoning due to the strong adsorption of polar organic molecules, such as the product generated here.^40,50^

**Fig. 4:**
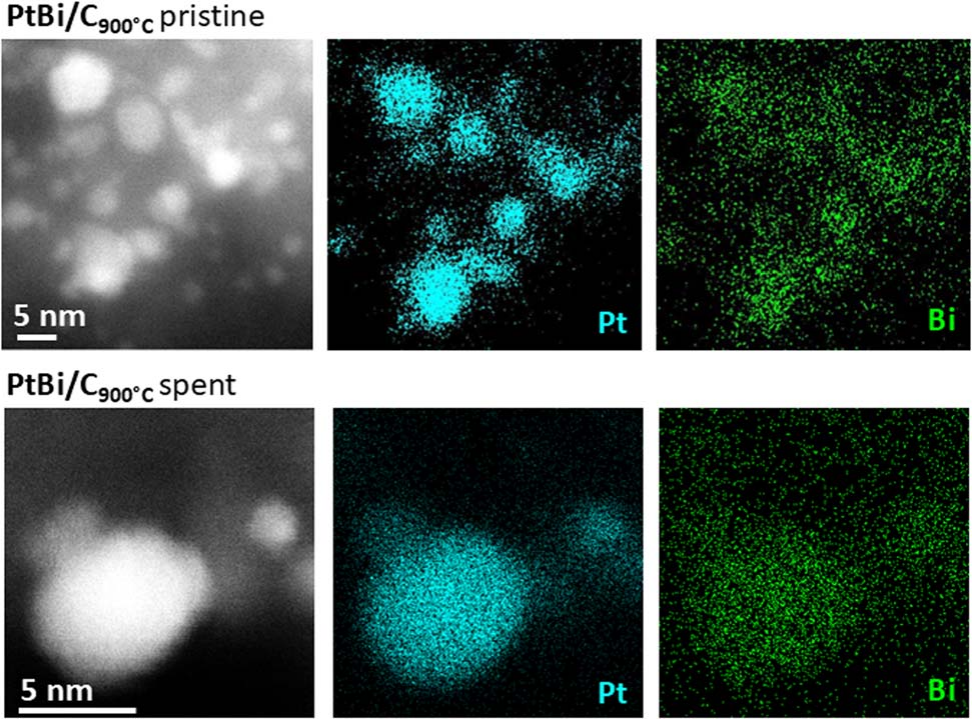
HAADF STEM image and EDX maps of the pristine (Top) and spent (Bottom) PtBi/C_900°C_. Spatial correlation of the Pt M and Bi M signals in the EDX maps suggests the preservation of the alloyed state.

We next investigate the spent catalysts to identify spectroscopic signatures, which could explain the enhanced activity of bimetallic PtBi/C catalysts. Noteworthy, STEM imaging (Fig. S13) reveals minimal changes in NP size and distribution of the bimetallic PtBi/C materials after catalysis (3.8 ± 1.0 nm *vs.* 3.5 ± 1.0 nm), in sharp contrast with the monometallic Pt/C catalysts, which exhibit an increase in NP size by ≥20% (see Fig. S6). This observation underscores the superior stability of the bimetallic catalysts against sintering, a crucial property to enable the reutilisation of the catalyst (*vide infra*). The enhanced resistance to sintering of PtBi/C compared to Pt/C can be attributed to the segregation of Pt atoms by Bi and the preferential oxidation of Bi under reaction conditions, resulting in Bi-rich surface layers or Pt–[O]_*x*_–Bi motifs that minimize sintering.^51^ Furthermore, STEM EDX analysis of the spent PtBi/C catalyst shows a spatial correlation of the Pt M and Bi M signals, indicating that the alloyed PtBi structure is preserved (see Fig. 4). These findings are further supported by CO chemisorption; the highly sintered Pt/C_600°C_ catalyst exhibits a higher CO uptake compared to the bimetallic PtBi/C_600°C_ (0.79 *vs.* 0.30 mmol CO per g Pt), confirming that Pt and Bi remain alloyed after catalysis. Note that after catalysis, the CO uptake capacity of the monometallic and bimetallic catalysts decreases by around 50% and 70% respectively, highlighting relevant changes in the NPs composition and/or structure. Furthermore, the bimetallic catalysts show prolonged desorption, suggesting reversible adsorption of CO-like intermediates. This process is not observed for the monometallic Pt/C counterparts, pointing to the irreversible adsorption and thus poisoning of surface sites by these intermediates. All spent catalysts exhibit a slight increase in *d*-spacing, likely due to oxygen incorporation and partial oxidation of the NPs (see SI 3.2 for details). Notably, the unusually large *d*-spacing of 0.39 nm observed in the pristine Pt/C_900°C_ disappeared after catalysis, suggesting the removal of carbon from the structure upon oxidation during catalysis. This observation further supports the presence of Pt carbide in the fresh catalyst. We also perform XAS on the spent catalysts. Analysis of the Bi L_2_ and L_3_ edges shows that Bi sites in both bimetallic catalysts are reduced to Bi(iii) (Fig. S20). Since no difference in Bi oxidation state is found between PtBi/C_600°C_ and PtBi/C_900°C_, we infer that the differences in catalytic performance arise from changes in the environment of the Pt centers. Examination of the Pt L_3_ edge (see SI 3.5.1) reveals a decrease in white line intensity after catalysis, indicating reduction of Pt under reaction conditions, in agreement with previous observations for PtBi/Al_2_O_3_ during benzyl alcohol oxidation.^40^ Notably, the catalysts prepared at lower temperatures exhibit a significant edge shift to lower energy. This phenomenon is most pronounced in the least-performing Pt/C_600°C_ catalyst, where the proportion of Pt(0) increases from 41% to 62%, as determined by linear combination fitting of the spectra with Pt(0) foil and [Pt(II)(COD)(OSi(O^*t*^Bu)_3_)_2_] reference spectra (see Table S15). In sharp contrast, PtBi/C_900°C_ shows only a moderate increase of Pt(0) (3% increase) sites. Overall, the large increase of Pt(0) sites is correlated with lower catalytic activity, whereas catalysts displaying higher conversion exhibit a smaller change in the oxidation state. These observations suggest that an excess of Pt(0) surface sites may promote stronger binding of coordinating species (*e.g.*, solvent molecules or CO-like intermediates), leading to site poisoning. These findings are consistent with substantially lower CO uptake of the more active bimetallic PtBi/C catalysts (*vide supra*). Thus, the presence of a large amount of Pt(0) surface sites can possibly be prevented by structural changes, such as the use of Bi as a promoter or the formation of larger particles with unusual morphology, as highlighted by the higher catalytic performance of Pt(Bi)/C_900°C_.

**Fig. 5:**
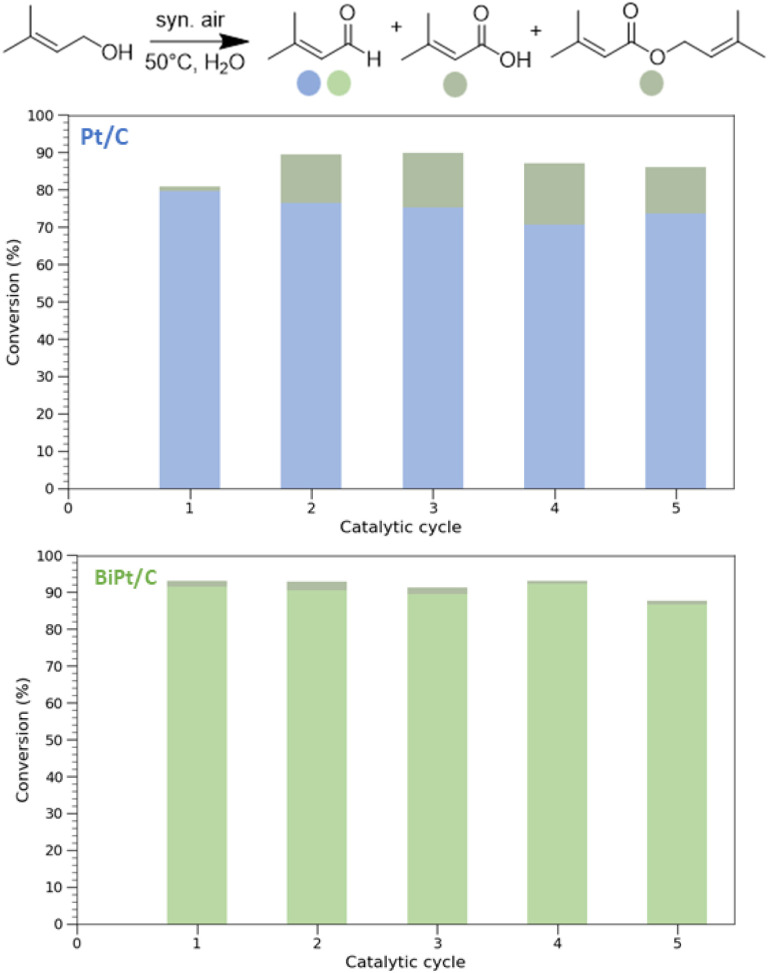
(Top) Schematic selective oxidation of prenol to prenal and related byproducts. (Bottom) Oxidation conversion of prenol using Pt/C_900°C_ (blue) and PtBi/C_900°C_ (green) over 5 catalytic cycles. The coloured part of the bar represents the conversion to prenal, while the grey part is the conversion to the corresponding acid and ester.

Finally, we evaluate the selective oxidation of prenol, a key intermediate in the industrial production of citral, which is commonly used in the perfumery industry, as a food additive, or as an intermediate for the synthesis of menthol, vitamin A, and vitamin E.^52–58^ We evaluate this reaction at 50 °C for 9 h with 5 V% of prenol in water and a catalyst loading corresponding to 0.2 mol% of Pt to prenol (see SI 4.2). Note that when 95 V% prenol in water is used, low conversion is observed with PtBi/C_900°C_ (see Table S20), highlighting the crucial role of H_2_O in this reaction. For Pt/C_900°C,_ we observe a conversion of 81% with a selectivity towards prenal of 96%. The conversion slightly increases (≈87%) upon recycling of the catalysts, while a drop in the selectivity is observed (around 80%), as shown in Fig. 5. In sharp contrast, the bimetallic PtBi/C_900°C_ catalyst shows an enhanced conversion (93%) and excellent selectivity (98%), as expected from its performance in ME oxidation. Notably, PtBi/C_900°C_ can be re-used multiple times with stable conversion and selectivity, highlighting the high stability of the catalyst. The superior recyclability of PtBi *versus* Pt underscores the role of Bi in promoting the stabilization of the catalyst, likely by inhibiting sintering and mitigating surface-poisoning effects. This highlights PtBi as a more robust and sustainable catalyst for repeated oxidation cycles.

## Conclusions

Well-dispersed and tailored PtBi NPs supported on carbon (PtBi/C) are prepared *via* SOMC, where the NPs' size can be tuned by the temperature during particle formation under H_2_. PtBi/C catalysts consist of alloyed NPs and exhibit excellent catalytic activity and stability for the selective aerobic oxidation of alcohols under mild aqueous conditions. Notably, larger PtBi NPs exhibit an enhanced performance in the selective oxidation of 2-methoxyethanol, a prototypical substrate, when compared to smaller NPs or the corresponding monometallic Pt NPs. The best-performing catalyst achieves 93% conversion and >98% selectivity in the oxidation of prenol—an industrially relevant intermediate—while maintaining a stable activity over multiple catalytic cycles. Furthermore, electron microscopy and X-ray absorption spectroscopy studies indicate that the presence of Bi regulates the Pt oxidation state, suppressing the presence of “excessive” Pt(0) surface species, which have previously been proposed to cause the site poisoning through strong binding of coordinating species, thereby improving activity and stability. The superior recyclability of PtBi/C compared to monometallic Pt/C further underscores the promotional role of Bi in stabilizing the NPs against sintering and preventing selectivity losses associated with larger NPs sizes. Overall, our findings provide valuable insights into the promotional effect of Bi through systematic comparison and the establishment of structure–activity relationships, thereby opening up new avenues for the rational design of robust, selective, and environmentally friendly oxidation catalysts.

## Author contributions

A. G. N. and C. C. conceptualized the study and were responsible for the methodology. A. G. N. performed the experiments, E. B. and M. P. performed the electron microscopy measurement. C. C. was responsible for supervision. A. G. N. and C. C. wrote the original draft. All authors edited the manuscript.

## Conflicts of interest

There are no conflicts to declare.

## Supplementary Material

SC-OLF-D5SC07190A-s001

## Data Availability

The data supporting this article have been included as part of the Supplementary Information (SI) and are openly accessible on Zenodo at 10.5281/zenodo.17433877. See DOI: https://doi.org/10.1039/d5sc07190a.
