# Supplementary material for: Selective aerobic oxidation of alcohols with supported Pt nanoparticles: effect of particle size and bismuth promotion
Source: Chem Sci. 2025 Oct 28;16(47):22603–10. doi: 10.1039/d5sc07190a (PMC12560347; doi:10.1039/d5sc07190a)
Supplement: SC-016-D5SC07190A-s001 [file SC-016-D5SC07190A-s001.pdf]

Supplementary Information (SI) for Chemical Science.
This journal is © The Royal Society of Chemistry 2025

Supporting Information

Selective Aerobic Oxidation of Alcohols with Supported Pt Nanoparticles: Effect of Particle Size and Bismuth Promotion

Anna Giorgia Nobile,[a] Enzo Brack,[a] Milivoj Plodinec,[a,b] Christophe Copéret[*,a]

[a] Department of Chemistry and Applied Biosciences, ETH Zurich, Vladimir-Prelog-Weg 2, 8093 Zurich, Switzerland.

[b] Scientific Center for Optical and Electron Microscopy (ScopeM), ETH Zurich, Otto-Stern-Weg 3, 8093 Zurich, Switzerland.

Summary

1. General considerations 3
2. Material preparation 5
   2.1. Support preparation 5
   2.2. Pt/C 5
   2.3. Bi/C 5
   2.4. PtBi/C 6
   2.5 PtBi/SiO_2 6
3. Characterization 8
   3.1. BET 8
   3.2. Microscopy 9
      3.2.1. Pt/C 10
      3.2.2. Bi/C 15
      3.2.3. PtBi/C cografting 16
      3.2.4. PtBi/SiO_2 21
   3.3. Chemisorption experiments 22
      3.3.1. H_2 chemisorption 22
      3.3.2. CO chemisorption 23
   3.4. CO-IR probe on SiO_2-supported materials 24
   3.5. X-Ray absorption spectroscopy 25
      3.5.1. Pt L_3-edge 25
      3.5.2. Bi L_3-edge 27
      3.5.3. Bi L_2-edge 28
4. Catalytic tests 29
   4.1. Experimental procedure 29
   4.2. Catalytic results 30
   4.3. ^{1}H NMR of catalytic tests 31

1. General considerations

Methods: All experiments were performed under an inert argon atmosphere using standard Schlenk, glovebox, and high vacuum techniques (≈10^{-5} mbar) unless mentioned otherwise. Pentane was dried by passage through double M-Braun SPS alumina solvent purification columns. Tetrahydrofuran (THF) and benzene were dried and freshly distilled under argon from purple Na^0/benzophenone. Deuterated benzene was vacuum distilled from Na^0/benzophenone. $[Bi(OSi(O^tBu)_3)_3]$[1], $[Pt(COD)(OSi(O^tBu)_3)_2]$[2], $[NaOSi(O^tBu)_3]$[3], and $[Mg(Bn)_2(THF)_2]$[4] were prepared according to literature procedures. Acticarbone was purchased from Calgon Carbon Company; all other reagents were purchased from Sigma-Aldrich or Strem Chemicals and used as received. Solution ^1H NMR spectra were recorded in Teflon J. Young valve-sealed NMR tubes on a Bruker 300 MHz spectrometer and the chemical shifts are reported in ppm and referenced to the respective solvent.

Brunauer–Emmett–Teller (BET): Nitrogen adsorption isotherms were recorded on a Bel-Mini apparatus (BEL Japan, Inc.) at 77 K. Approximately 100 mg of the sample were loaded into the cells. Prior to the adsorption measurements, the samples (transferred from the glovebox) were evacuated under vacuum (ca. 10^{-3} mbar) at RT for 1 h. Data was fitted according to the BET theory to obtain the specific surface area.

Elemental analysis: All elemental analysis measurements were performed by Mr. Peter Kälin at the Mikrolabor Service of ETH Zurich, or the Mikroanalytisches Labor Pascher (Pulvermühle 1, D-53424 Remagen, Germany).

H_2 Chemisorption: H_2-Chemisorption measurements were performed using a BELSORP MAX X gas and vapor adsorption analyzer from Microtrac. Ca. 70 mg of the sample was transferred to the sample cell under inert gas. First, a pre-treatment consisting of evacuation for 30 min at 150 °C was performed. Then, the H_2 uptake was measured at 25 °C. The uptake for generating a monolayer of CO was determined as the axis intercept from interpolating the points after the initial jump with a linear regression between p_1=5 kPa and p_2=40 kPa.

CO Chemisorption: CO-Chemisorption measurements were performed using a BELSORP MAX X gas and vapor adsorption analyzer from Microtrac. For the pristine materials, ca. 100 mg of sample was transferred air-free directly after H_2 treatment to the sample cell; for the spent materials, ca. 100 mg of sample was first dried in air at 120 °C, followed by a vacuum treatment at 120 °C for 6 h. All samples then underwent a pre-treatment consisting of evacuation for 30 min at 150 °C. In the next step, the CO uptake was measured at 25 °C. The uptake for generating a monolayer of CO was determined as the axis intercept from interpolating the points after the initial jump with a linear regression between p_1=5 kPa and p_2=45 kPa.

CO-IR probe: Regarding the IR studies upon CO adsorption (CO-IR probe), the IR spectra were recorded from freshly prepared, self-supporting pellets (11-14 mg) of the studied

materials. Self-holding pellets on an aluminum ring were placed inside an IR shuttle (glass vacuum cell) equipped with CaF_2 windows. The cell was first evacuated under high vacuum (10^{-5} mbar), and a blank spectrum of the material, prior to CO addition, was measured. After exposure to CO gas, another spectrum was recorded. A third spectrum was collected after evacuation at 10^{-5} mbar for 5 minutes to investigate the disappearance of the CO bands from the reversibly bound CO molecules. For discussion, the background-subtracted IR spectrum is shown.

Electron Microscopy:

Transmission electron microscopy (TEM) and scanning TEM (STEM) measurements were recorded on a double CS-corrected JEOL JEM-ARM300F Grand ARM "Vortex" (scanning) transmission electron microscope operated at accelerating voltages of 200 or 300 kV. The samples were dry casted as a solid onto ultrathin carbon on lacey carbon 400 mesh copper grids (TedPella) in an Argon glovebox and mounted on a vacuum transfer holder (GATAN). The spent catalysts were filtered and dried overnight at 200 °C before being analyzed. STEM energy dispersive X-ray spectroscopy (STEM-EDX) maps were acquired using a dual SDD EDX detector system (each with a 100 mm^2 active area), providing a total solid angle of 1.6 sr and an energy resolution of ≤ 133 eV.

Scanning electron microscopy EDX (SEM–EDX) mapping was carried out in a TFS Quattro ESEM operated at 20 kV, equipped with an EDAX Octane Elite 70 mm^2 detector. Secondary electron (SE) images were acquired using an Everhart–Thornley detector (ETD). **Bi/C** samples are first dispersed in isopropanol by ultrasonication and then drop-cast onto an alumina holder for SEM analysis.

2. Material preparation

2.1. Support preparation

Carbon functionalization: Acticarbone (3 g) was calcined under air at 500 °C for 1 h,[5] followed by drying under high vacuum at 200 °C for 12 h.

Titration of reactive functionalities: A solution of [Mg(Bn)$_2$(THF)$_2$] (ca. 15 mg) in C_6D_6 was slowly added to a suspension of carbon (ca. 50 mg) while stirring. The resulting mixture was stirred for 24 hours, after which a ferrocene (ca. 5 mg, 0.03 mmol) solution was added and a ^{1}H solution NMR spectrum measured (recycle delay d1 = 60 s).

Before calcination: 0.073 mmol/g

After calcination: 0.17 mmol/g

Elemental analysis after calcination: 85.03 wt% C, 9,71 wt% O, 1.26 wt% H, 1.11 wt% N.

2.2. Pt/C

A solution of [Pt(COD)(OSi(O^{t}Bu)$_3$)$_2$] (65.5 mg, 0.079 mmol, 1 eq. to surface reactive groups) in benzene was slowly added to a suspension of mesoporous carbon (464.0 mg) while stirring. The resulting mixture was stirred for 12 hours, the carbon was then filtered off, washed with benzene (2 x 5 mL) and pentane (5 mL). The combined benzene washing solution was collected and analyzed by ^{1}H NMR spectroscopy in C_6D_6 using ferrocene as an internal standard to quantify the remaining precursors during the grafting step. The material was dried under high vacuum for 2h at RT. The material was subjected to a hydrogen flow treatment at 600 °C (300 °C/h ramp) and 1 bar of hydrogen for 12 hours, followed by evacuation under high vacuum and storage in a glovebox to give carbon-supported Pt nanoparticles **Pt/C$_{600°C}$**. **Pt/C$_{600°C}$** was then treated under hydrogen flow (1 bar) at 750°C or 900°C for 12 h (300 °C/h ramp) to give **Pt/C$_{750°C}$** and **Pt/C$_{900°C}$**, respectively.

Elemental analysis **Pt/C$_{600°C}$**: 3.02 wt% Pt

Elemental analysis **Pt/C$_{750°C}$**: 4.12 wt% Pt

Elemental analysis **Pt/C$_{900°C}$**: 5.27 wt% Pt

2.3. Bi/C

A solution of [Bi(OSi(O^{t}Bu)$_3$)$_3$] (52.8 mg, 0.0529 mmol, 1 eq. to surface reactive groups) in benzene was slowly added to a suspension of mesoporous carbon (311.2 mg) while stirring. The resulting mixture was stirred for 72 hours, the carbon was then filtered off, washed with benzene (2 x 5 mL) and pentane (5 mL). The combined benzene washing

solution was collected and analyzed by ^1H NMR spectroscopy in C_6D_6 using ferrocene as an internal standard to quantify the remaining precursors during the grafting step. The material was dried under high vacuum for 2h at RT. The material was subjected to a hydrogen flow treatment at 600 °C (300 °C/h ramp) and 1 bar of hydrogen for 12 hours, followed by evacuation under high vacuum and storage in a glovebox to give carbon-supported Bi nanoparticles **Bi/C$_{600°C}$**.

Elemental analysis: 2.04 wt% Bi

2.4. PtBi/C

A solution of [Pt(COD)(OSi(O^{t}Bu)$_3$)$_2$] (60.4 mg, 0.073 mmol, 0.7 eq. to surface reactive groups) in benzene was slowly added to a suspension of mesoporous carbon (604.8 mg) while stirring. The resulting mixture was stirred for 7 hours, followed by the addition of a solution of [Bi(OSi(O^{t}Bu)$_3$)$_3$] (34.4 mg, 0.0345 mmol, 0.3 eq. to surface reactive groups) in benzene and 72 h of stirring. The carbon was then filtered off, washed with benzene (2 x 5 mL) and pentane (5 mL). The combined benzene washing solution was collected and analyzed by ^1H NMR spectroscopy in C_6D_6 using ferrocene as an internal standard to quantify the remaining precursors during the grafting step. The material was dried under high vacuum for 2h at RT. The material was subjected to a hydrogen flow treatment at 600 °C (300 °C/h ramp) and 1 bar of hydrogen for 12 hours, followed by evacuation under high vacuum (10^{-5} mbar) and storage in a glovebox to give carbon-supported Pt nanoparticles **PtBi/C$_{600°C}$**. **PtBi/C$_{600°C}$** was then treated under hydrogen flow (1 bar) at 750°C or 900°C for 12 h (300 °C/h ramp) to give **PtBi/C$_{750°C}$** and **PtBi/C$_{900°C}$**, respectively.

Elemental analysis **PtBi/C$_{600°C}$**: 3.42 wt% Pt, 1.30 wt% Bi

Elemental analysis **PtBi/C$_{750°C}$**: 3.08 wt% Pt, 1.17 wt% Bi

Elemental analysis **PtBi/C$_{900°C}$**: 2.50 wt% Pt, 0.80 wt% Bi

2.5 PtBi/SiO_2

Preparation of silica support: Silica support, partially dehydroxylated at 700 °C (**SiO$_{2\text{-}700°C}$**), was prepared by heating Degauss Aerosil (204 m^2/g) to 500 °C (5 °C/min), calcination in air for 8 hours, evacuation at high vacuum (10^{-5} mbar), followed by maintaining a temperature of 500 °C for 8 hours, heating to 700 °C (1 °C/min), and maintaining 700 °C for 20 hours. ^1H NMR titration of the **SiO$_{2\text{-}700°C}$** using [Mg(Bn)$_2$(THF)$_2$] yielded 0.26 - 0.28 mmol OH g^{-1} corresponding to 0.8 accessible OH groups per nm^2.

Preparation of supported **PtBi/SiO$_{2\text{-}600°C}$** nanoparticles: In a 50 ml Schlenk flask, a transparent solution of [Bi(OSi(O^{t}Bu)$_3$)$_3$] (1 eq. to surface OH groups) in benzene was slowly added to a benzene (3 mL) suspension of **SiO$_{2\text{-}700°C}$** (about 200 mg) at room temperature inside an argon-filled glovebox. After stirring for 72 h at room temperature,

the solid was washed with benzene (3 x 2 mL) and pentane (1 x 2 mL) and dried under high vacuum (10^{-5} mbar) at room temperature for 2 hours. The material was subjected to a synthetic air (SA) flow treatment at 600 °C (300 °C/h ramp) and 1 bar of SA for 12 hours, followed by evacuation under high vacuum to give **$Bi/SiO_{2-600°C}$**. In the next step, a transparent solution of $[Pt(COD)(OSi(O^tBu)_3)_2]$ (1 eq. to surface OH groups) in benzene was slowly added to a benzene (3 mL) suspension of **$Bi/SiO_{2-600°C}$**. After stirring for 12 h at room temperature, the solid was washed with benzene (3 x 2 mL) and pentane (1 x 2 mL) and dried under high vacuum (10^{-5} mbar) at room temperature for 2 hours. The combined benzene washing solution was collected and analyzed by ^1H NMR spectroscopy in C_6D_6 using ferrocene as an internal standard to quantify the left precursors during the grafting step. The material was subjected to a hydrogen flow treatment at 600 °C (300 °C/h ramp) and 1 bar of hydrogen for 12 hours, followed by evacuation under high vacuum (10^{-5} mbar) and storage in a glovebox to give silica-supported PtBi nanoparticles **$PtBi/SiO_{2-600°C}$**.

Elemental analysis **$PtBi/SiO_{2-600°C}$**: 3.48 wt% Pt, 2.25 wt% Bi

3. Characterization

3.1. BET

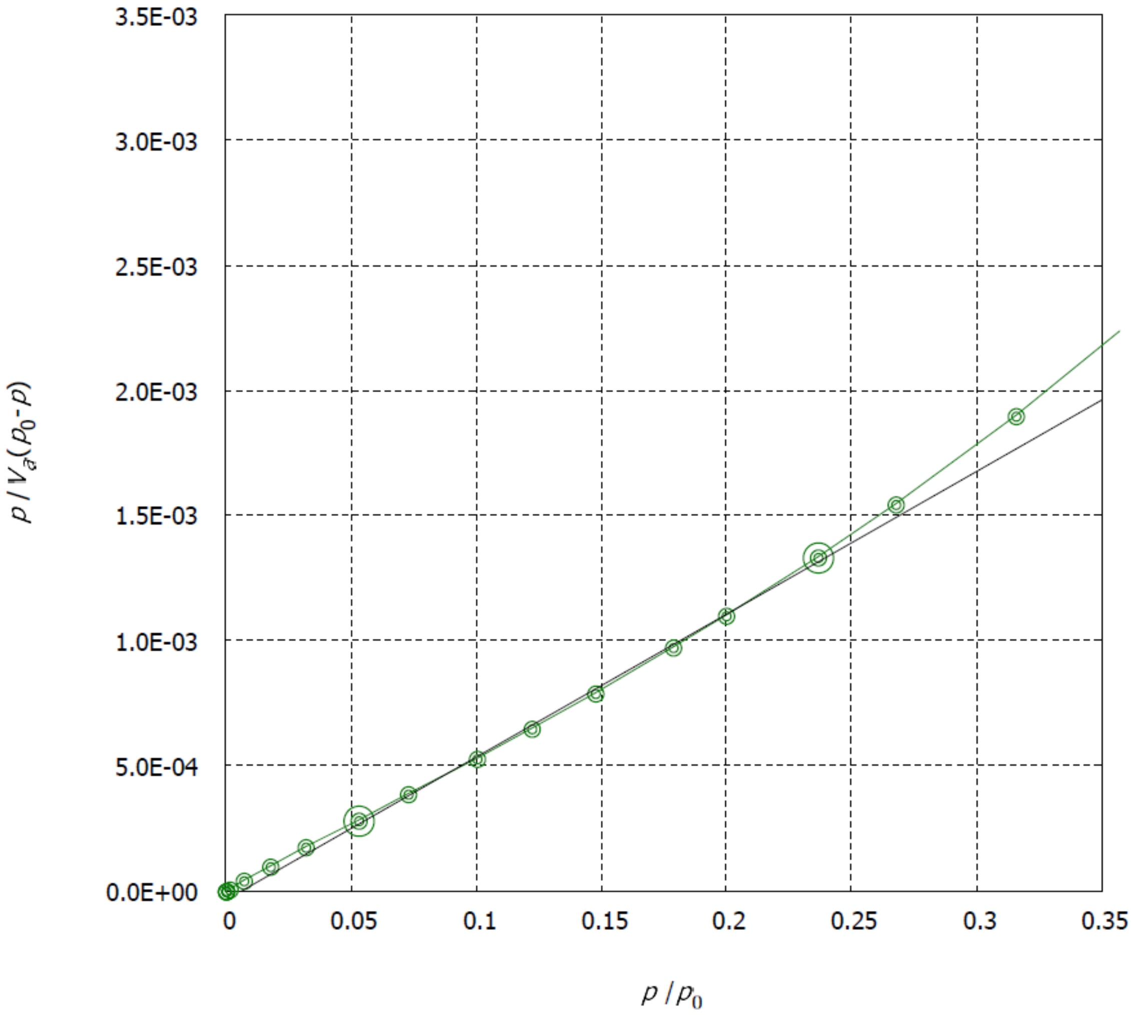

Figure S1. N_2 Adsorption isotherms of the carbon support after calcination at 500 °C.

Surface area: $a_{s,BET}$ = 766.69 m^2/g

Total pore volume: V_p = 0.5935 cm^3/g

Average pore diameter: d_p = 1.5483 nm

3.2. Microscopy

Table S1. Summary of the particle sizes distributions and observed d-spacings of the prepared pristine and spent materials.

	Particle size (nm)	d spacing (nm)
**Pt/C$_{600°C}$**	2.44 ± 0.73	0.2077, 0.1809, 0.2332
**Pt/C$_{750°C}$**	3.73 ± 2.47	
**Pt/C$_{900°C}$**	10.86 ± 9.77	0.3978, 0.2942
**PtBi/C$_{600°C}$**	1.57 ± 0.51	0.1911, 0.1806
**PtBi/C$_{750°C}$**	2.65 ± 1.19	
**PtBi/C$_{900°C}$**	3.86 ± 1.03	0.2203, 0.1899
**Pt/C$_{600°C}$** spent	3.31 ± 1.23	0.1919, 0.2138, 0.2202
**Pt/C$_{900°C}$** spent	12.97 ± 11.40	0.2544, 0.2292, 0.1891
**PtBi/C$_{600°C}$** spent	1.71 ± 0.70	0.1863, 0.2054, 0.2253
**PtBi/C$_{900°C}$** spent	3.54 ± 1.05	0.2657, 0.2054, 0.1613
**PtBi/SiO$_{2\text{-}600°C}$**	2.86 ± 0.50	

3.2.1. Pt/C

Pt/C$_{600°C}$ pristine

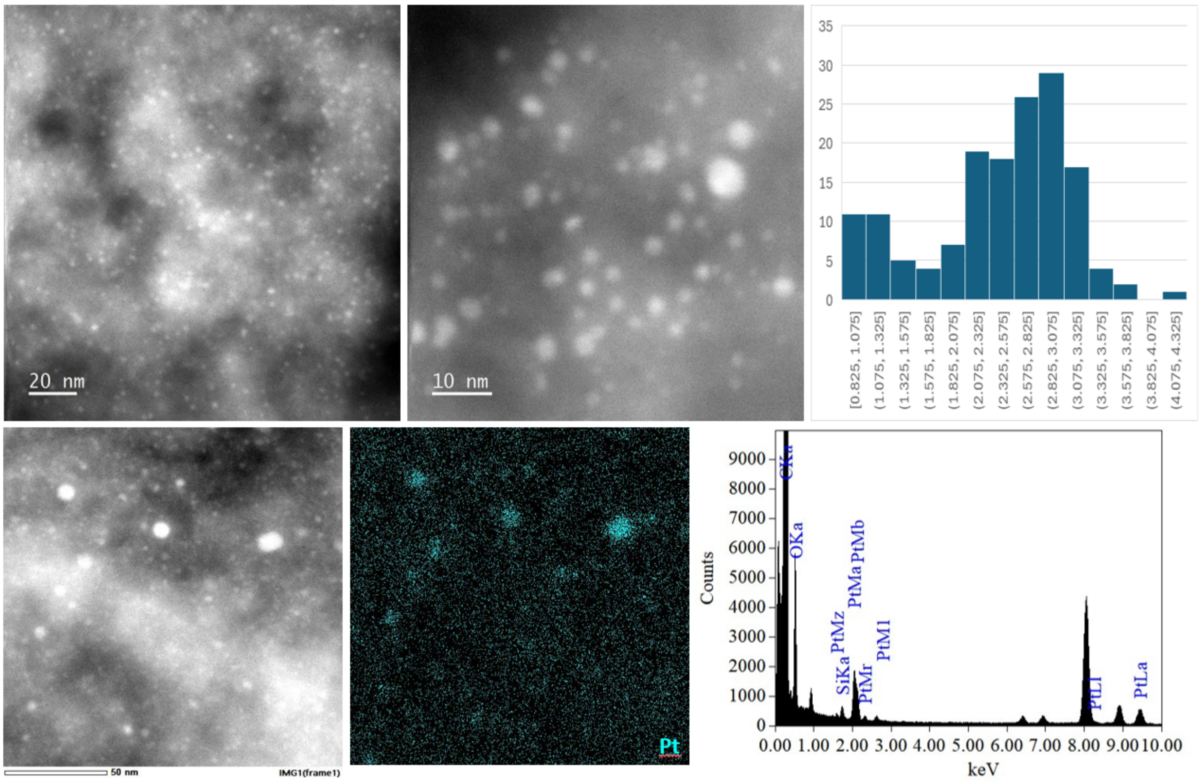

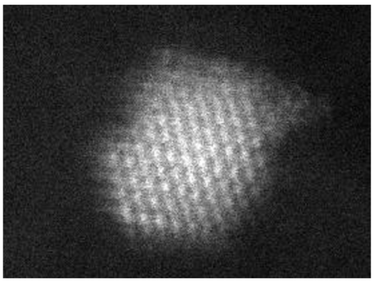

d-spacings (nm): 0.2077, 0.1809, 0.2332

*Figure S2. Top: Representative HAADF STEM images (left) and particle size distribution (right) of **Pt/C$_{600°C}$** pristine. Middle: HAADF STEM image and EDX map (left) and spectrum (right) of **Pt/C$_{600°C}$** pristine. Bottom: High magnification HAADF STEM image for d-spacing analysis of **Pt/C$_{600°C}$**.*

*Table S2. Quantification of the STEM EDX spectrum of **Pt/C$_{600°C}$** pristine.*

Element	Mass%	Atom%
C K	95.89	98.26
Pt M	1.70	0.11
O K	1.75	1.35
Si K	0.66	0.29

$Pt/C_{600°C}$ spent

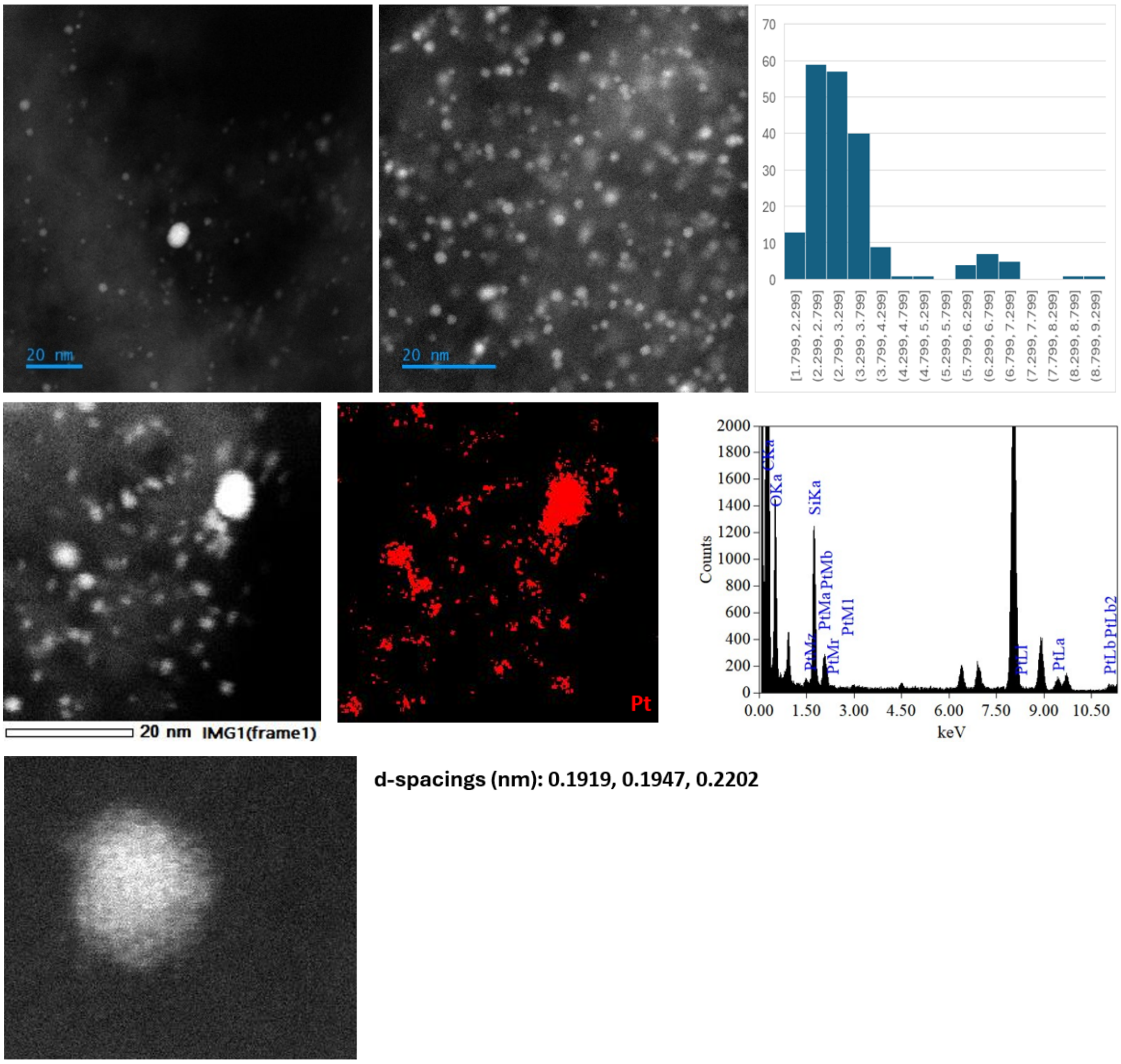

*Figure S3. Top: Representative HAADF STEM images (left) and particle size distribution (right) of **$Pt/C_{600°C}$** spent. Middle: HAADF STEM EDX maps (left) and spectrum (right) of **$Pt/C_{600°C}$** spent. Bottom: High magnification HAADF STEM image for d-spacing analysis of **$Pt/C_{600°C}$** spent.*

*Table S3. Quantification of the STEM EDX spectrum of **$Pt/C_{600°C}$** spent.*

	Mass%	Atom%
C K	91.35	95.76
Pt M	2.31	0.15
O K	3.70	2.91
Si K	2.64	1.19

Pt/$C_{750°C}$ pristine

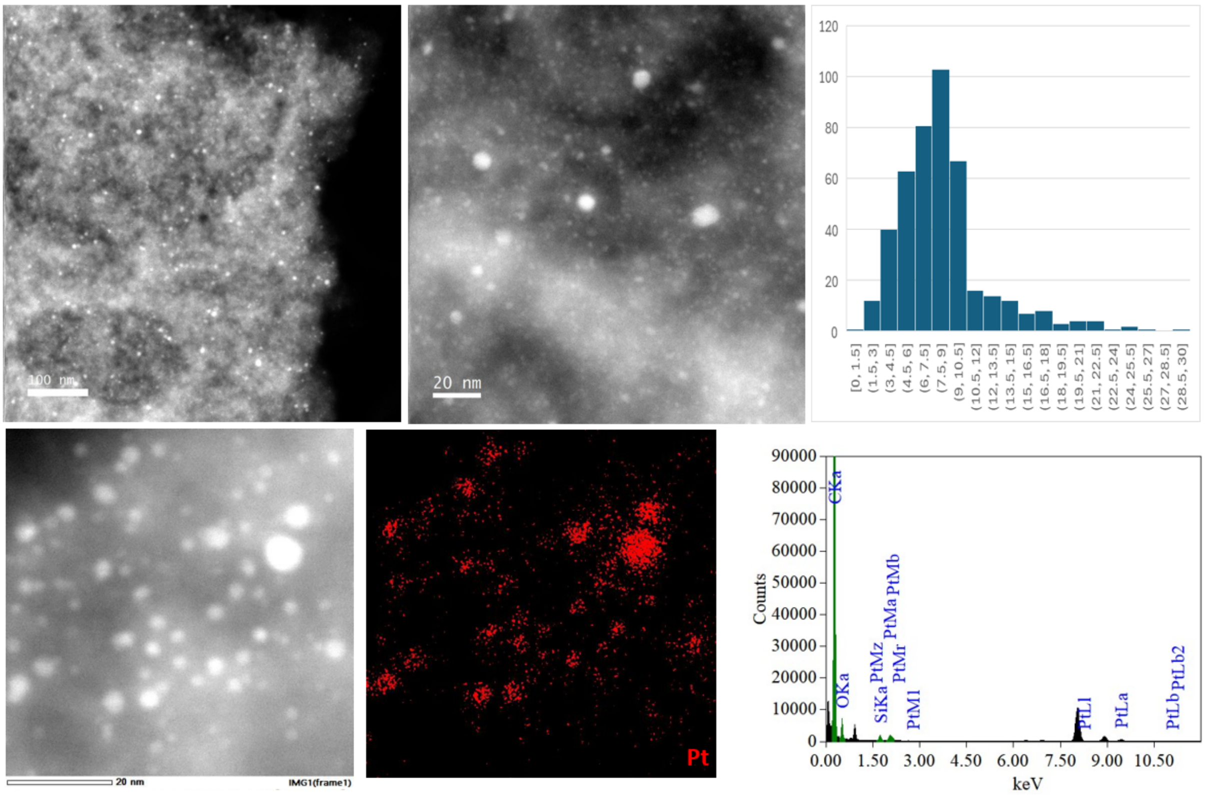

*Figure S4. Top: Representative HAADF STEM images (left) and particle size distribution (right) of **Pt/$C_{750°C}$** pristine. Bottom: HAADF STEM EDX maps (left) and spectrum (right) of **Pt/$C_{750°C}$** pristine.*

*Table S4. Quantification of the STEM-EDX spectrum of **Pt/$C_{750°C}$** pristine.*

Element	Mass%	Atom%
C K	95.82	98.53
Pt M	2.048	0.15
O K	1.54	1.19
Si K	0.29	0.13

Pt/C$_{900°C}$ pristine

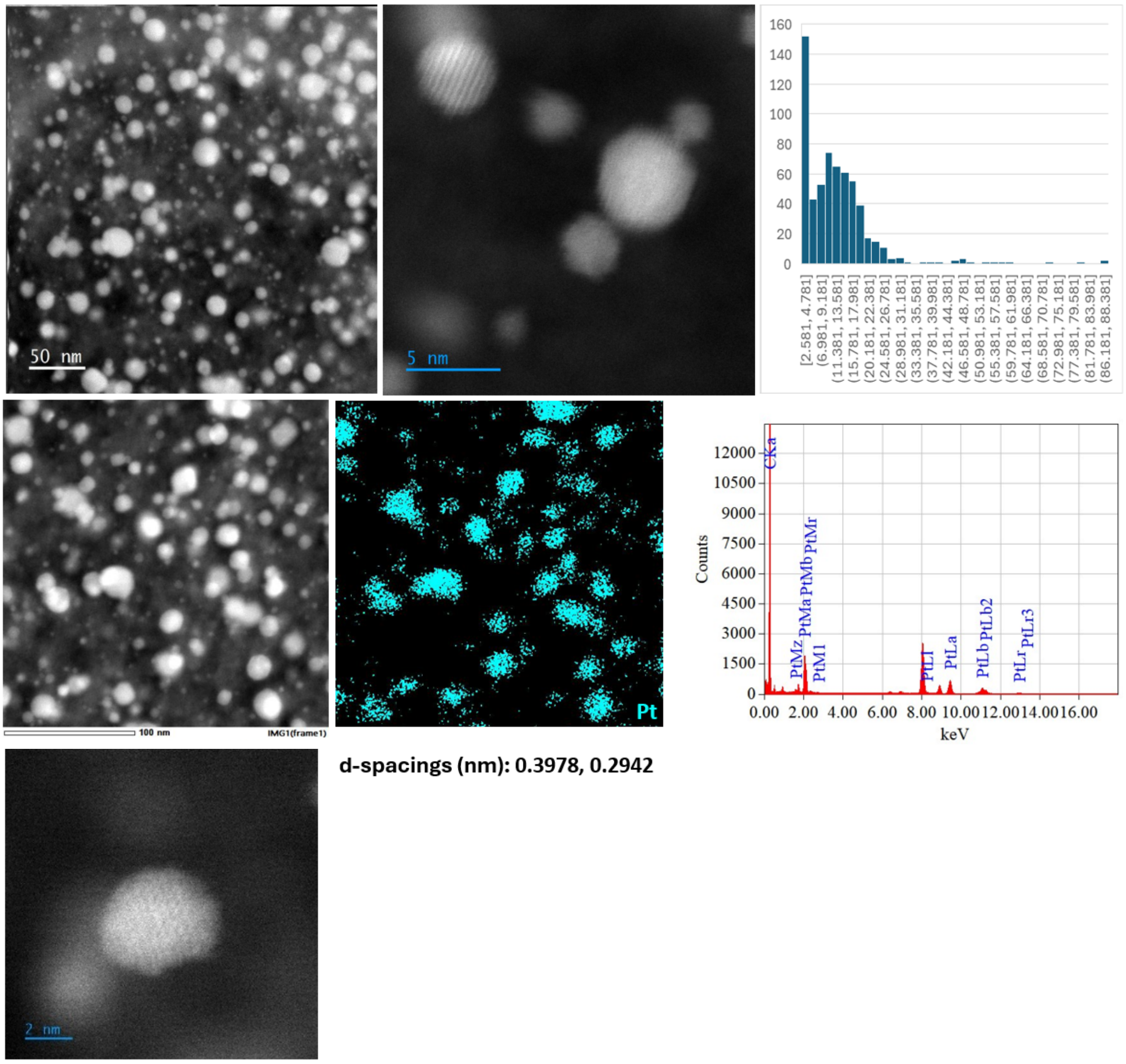

*Figure S5. Top: Representative HAADF STEM images (left) and particle size distribution (right) of **Pt/C$_{900°C}$** pristine. Middle: HAADF STEM EDX maps (left) and spectrum (right) of **Pt/C$_{900°C}$** pristine. Bottom: High magnification HAADF STEM image for d-spacing analysis of **Pt/C$_{900°C}$** pristine.*

*Table S5. Quantification of STEM EDX spectrum of **Pt/C$_{900°C}$** pristine.*

Element	Mass%	Atom%
C K	80.82	98.56
Pt M	19.18	1.44

Pt/C$_{900^{\circ}C}$ spent

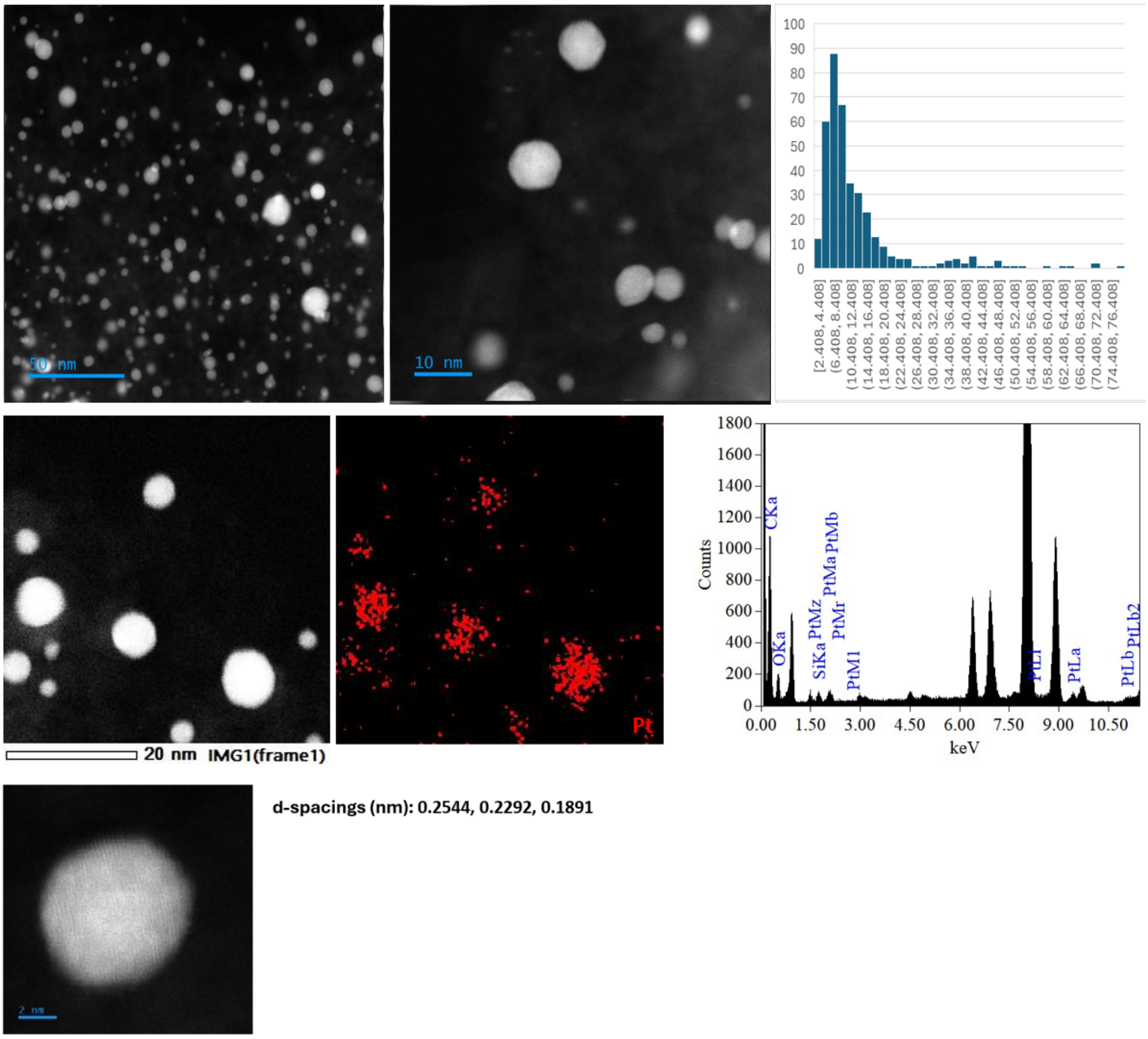

*Figure S6. Top: Representative HAADF STEM images (left) and particle size distribution (right) of **Pt/C$_{900^{\circ}C}$** spent. Middle: HAADF STEM EDX maps (left) and spectrum (right) of **Pt/C$_{900^{\circ}C}$** spent. Bottom: High magnification HAADF STEM image for d-spacing analysis of **Pt/C$_{900^{\circ}C}$** spent.*

*Table S6. Quantification of STEM EDX spectrum of **Pt/C$_{900^{\circ}C}$** spent.*

Element	Mass%	Atom%
C K	83.59	94.37
Pt M	9.77	0.68
O K	4.78	4.05
Si K	1.86	0.90

3.2.2. Bi/C

Bi/C$_{600°C}$

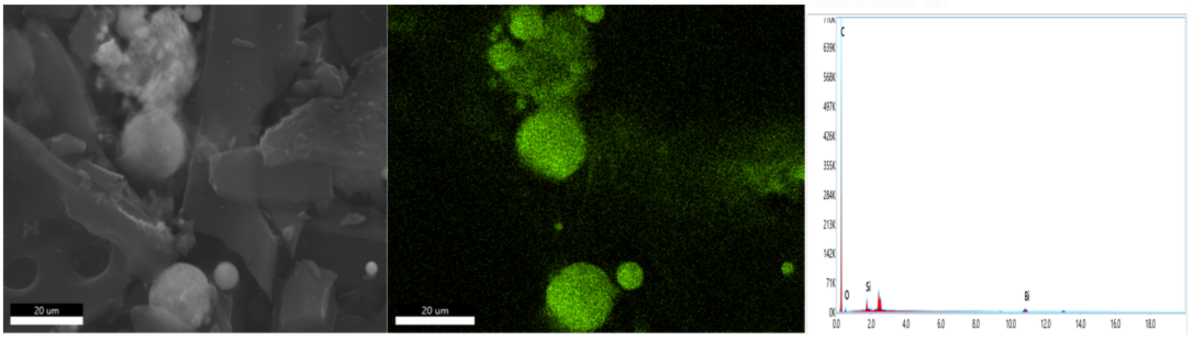

*Figure S7. SE- SEM image of **Bi/C$_{600°C}$** (left), SEM EDX map (center) and spectrum (right).*

*Table S7. Quantification of STEM EDX spectrum from Fig. S7 of **Bi/C$_{600°C}$** pristine.*

Element	Mass%	Atom%
C K	86.1	96.3
Bi M	9.7	0.6
O K	3.0	2.5
Si K	1.3	0.6

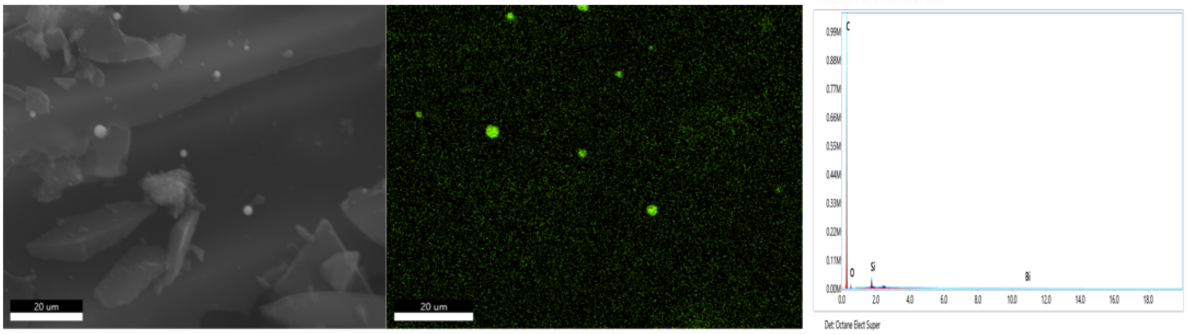

*Figure S8: SE-SEM image (left) of **Bi/C$_{600°C}$**, SEM EDX map (center) and spectrum (right).*

*Table S8 Quantification of STEM EDX spectrum from Fig. S8 of **Bi/C$_{600°C}$** pristine.*

Element	Mass%	Atom%
C K	93.8	96.3
Bi M	0.9	0.1
O K	4.1	3.1
Si K	1.2	0.5

3.2.3. PtBi/C cografting

PtBi/C$_{600°C}$ pristine

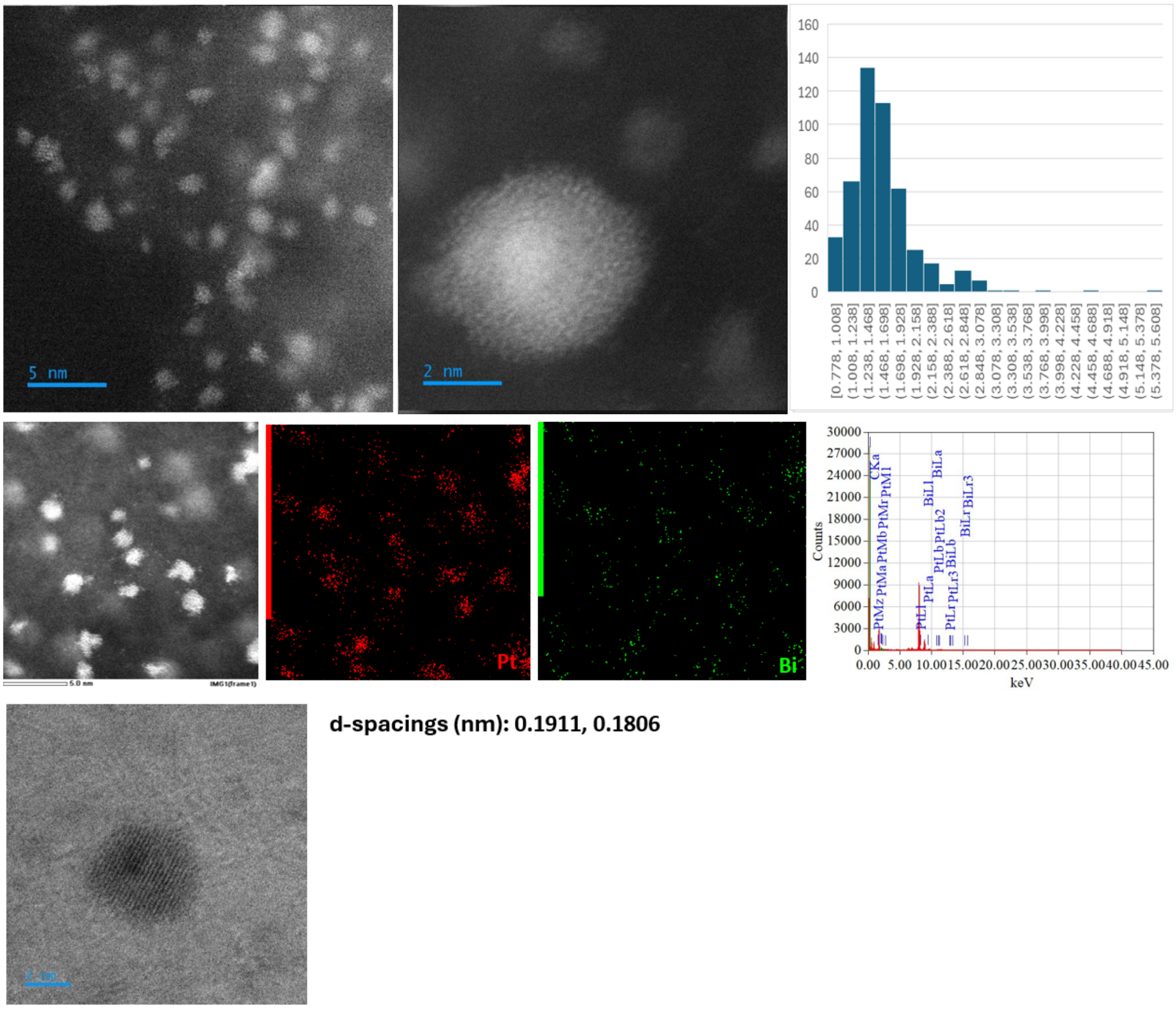

Figure S9. Top: Representative HAADF STEM images (left) and particle size distribution (right) of ***PtBi/C$_{600°C}$*** *pristine. Middle: HAADF STEM EDX maps (left) and spectrum (right) of* ***PtBi/C$_{600°C}$*** *pristine. Bottom: High magnification ABF STEM image for d-spacing analysis of* ***PtBi/C$_{600°C}$*** *pristine.*

Table S9. Quantification of STEM EDX spectrum of ***PtBi/C$_{600°C}$*** *pristine.*

Element	Mass%	Atom%
C K	95.96	99.75
Pt M	3.05	0.20
Bi M	0.99	0.06

PtBi/C$_{600°C}$ spent

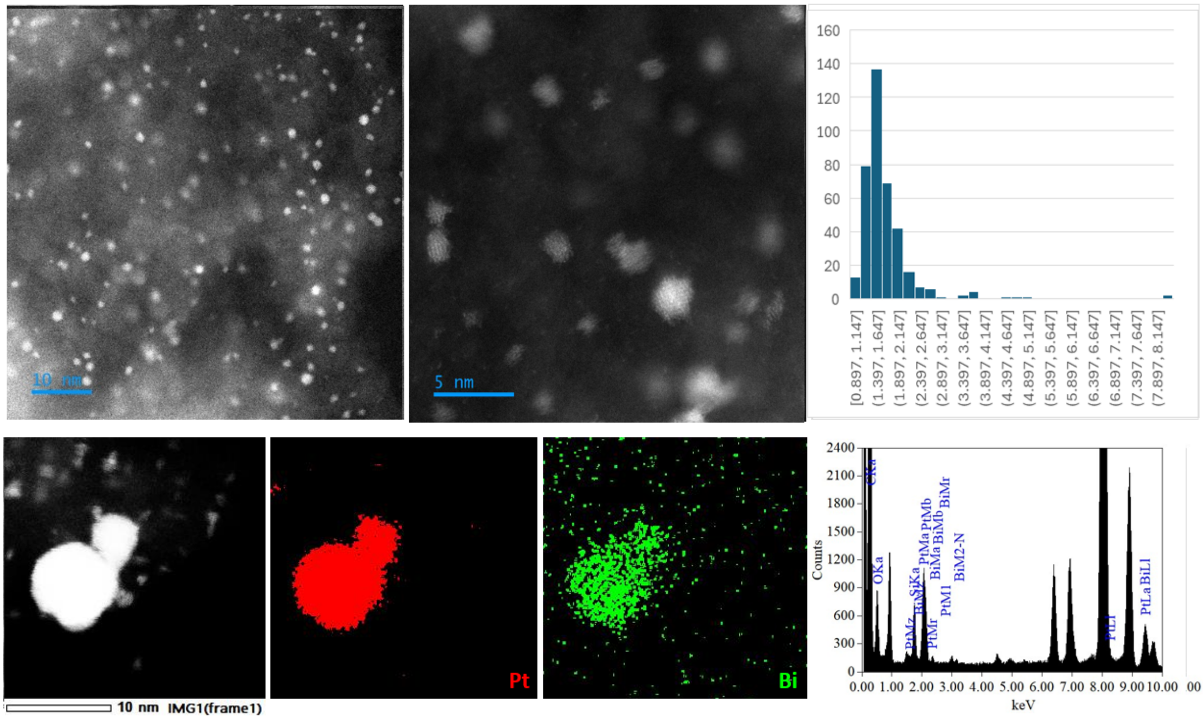

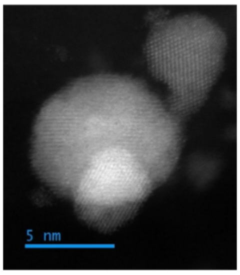

d-spacings (nm): 0.2054, 0.2253, 0.1863

*Figure S10. Top: Representative HAADF STEM images (left) and particle size distribution (right) of **PtBi/C$_{600°C}$** spent. Middle: HAADF STEM EDX maps (left) and spectrum (right) of **PtBi/C$_{600°C}$** spent. Bottom: High magnification HAADF STEM image for d-spacing analysis of **PtBi/C$_{600°C}$** spent.*

*Table S10. Quantification of STEM EDX spectrum of **PtBi/C$_{600°C}$** spent.*

Element	Mass%	Atom%
C K	95.54	98.53
Pt M	2.20	0.14
Bi M	0.06	0.00
O K	1.08	0.83
Si K	1.11	0.49

PtBi/$C_{750°C}$ pristine

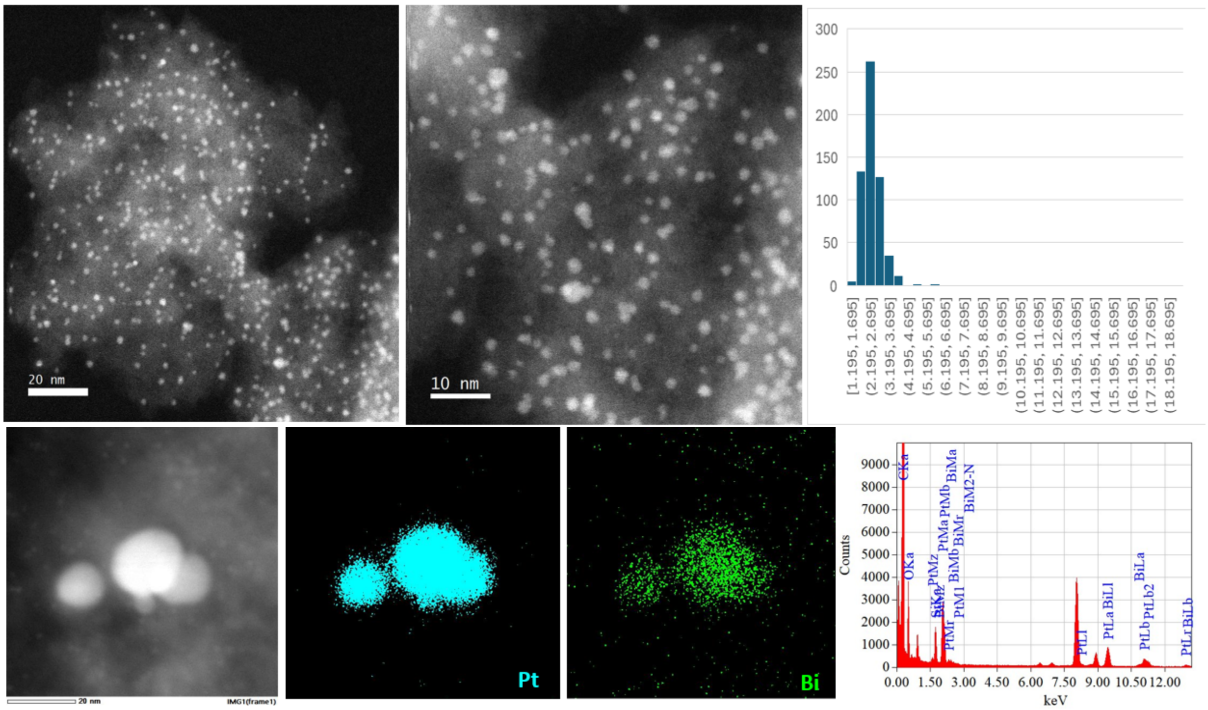

*Figure S11. Top: Representative HAADF STEM images (left) and particle size distribution (right) of **PtBi/$C_{750°C}$** pristine. Bottom: HAADF STEM EDX maps (left) and spectrum (right) of **PtBi/$C_{750°C}$** pristine.*

*Table S11. Quantification of STEM EDX spectrum of **PtBi/$C_{750°C}$** pristine.*

Element	Mass%	Atom%
C K	90.39	97.32
Pt M	5.84	0.39
Bi M	0.37	0.02
O K	2.04	1.65
Si K	1.37	0.63

PtBi/C$_{900°C}$ pristine

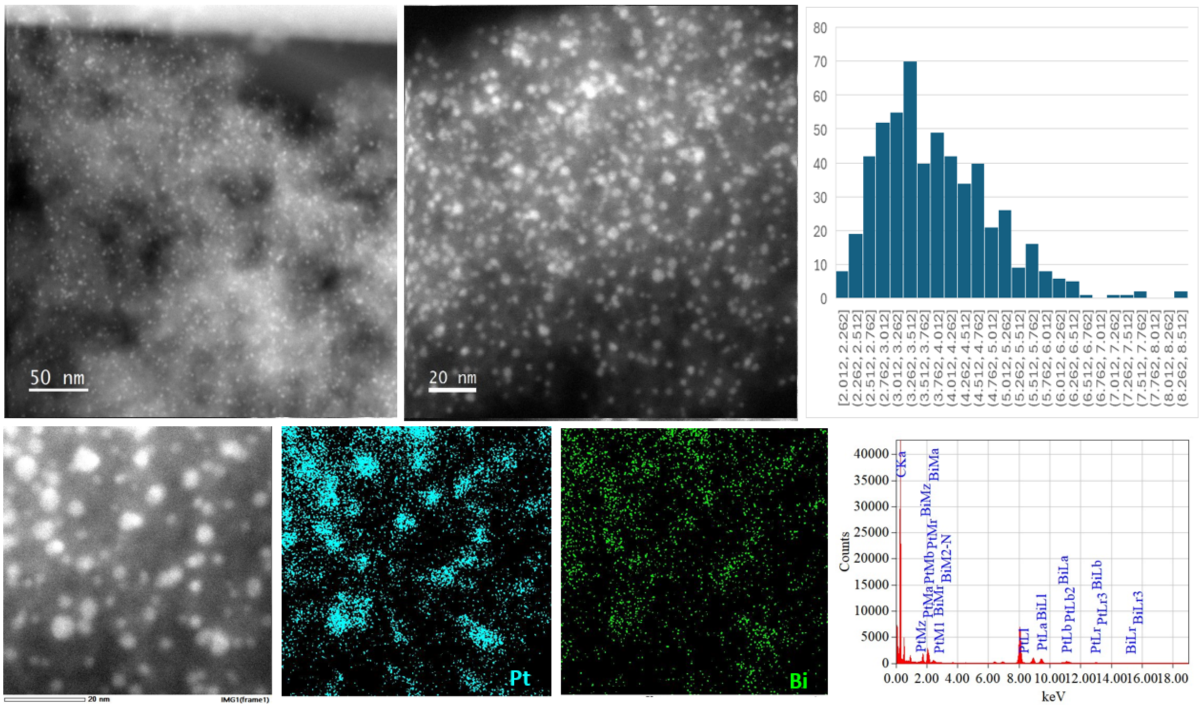

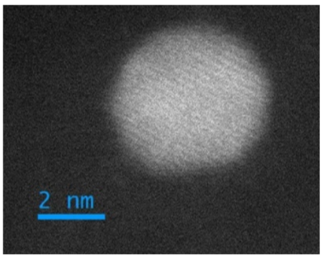

d-spacings (nm): 0.2203, 0.1899

*Figure S12. Top: Representative HAADF STEM images (left) and particle size distribution (right) of **PtBi/C$_{900°C}$** pristine. Middle: HAADF STEM EDX maps (left) and spectrum (right) of **PtBi/C$_{900°C}$** pristine. Bottom: High magnification HAADF STEM image for d-spacing analysis of **PtBi/C$_{900°C}$** pristine.*

*Table S12. Quantification of STEM EDX spectrum of **PtBi/C$_{900°C}$** pristine.*

Element	Mass%	Atom%
C K	84.14	95.37
Pt M	9.73	0.68
Bi M	0.10	0.01
O K	2.80	2.38
Si K	3.23	1.57

PtBi/C$_{900°C}$ spent

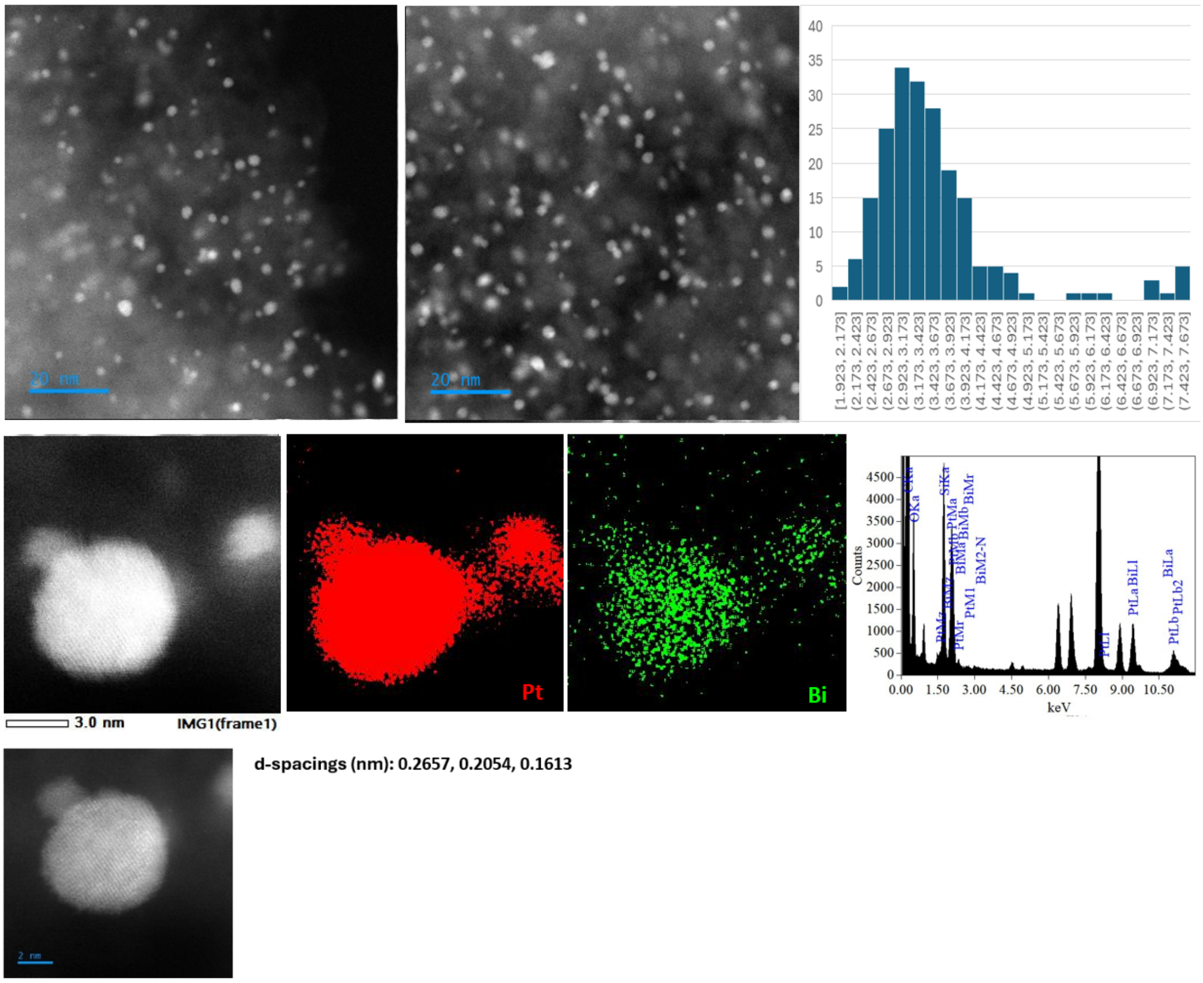

Figure S13. Top: Representative HAADF STEM image (left) and particle size distribution (right) of ***PtBi/C$_{900°C}$*** *spent. Middle: HAADF STEM EDX maps (left) and spectrum (right) of* ***PtBi/C$_{900°C}$*** *spent. Bottom: High magnification HAADF STEM image for d-spacing analysis of* ***PtBi/C$_{900°C}$*** *spent.*

Table S13. Quantification of STEM EDX spectrum of ***PtBi/C$_{900°C}$*** *spent.*

Element	Mass%	Atom%
C K	85.03	95.38
Pt M	7.47	0.52
Bi M	1.92	0.12
O K	3.60	3.03
Si K	1.98	0.95

3.2.4. PtBi/SiO_2

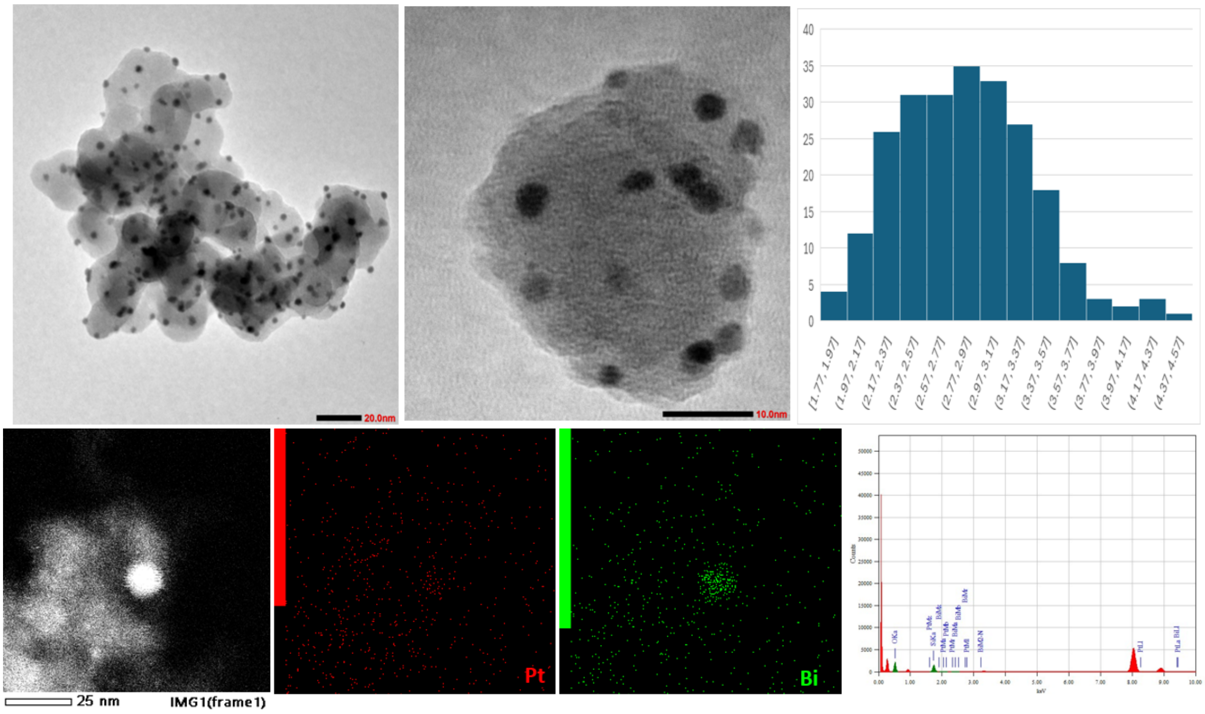

*Figure S14. Top: Representative ABF STEM images (left) and particle size distribution (right) of **PtBi/$SiO_{2\text{-}600°C}$**. Bottom: HAADF STEM EDX maps (left) and spectrum (right) of **PtBi/$SiO_{2\text{-}600°C}$**.*

*Table S14. Quantification of STEM EDX spectrum of **PtBi/$SiO_{2\text{-}600°C}$**.*

Element	Mass%	Atom%
Pt M	4.98	0.54
Bi M	0.66	0.07
O K	50.22	66.24
Si K	44.13	33.16

3.3. Chemisorption experiments

3.3.1. H_2 chemisorption

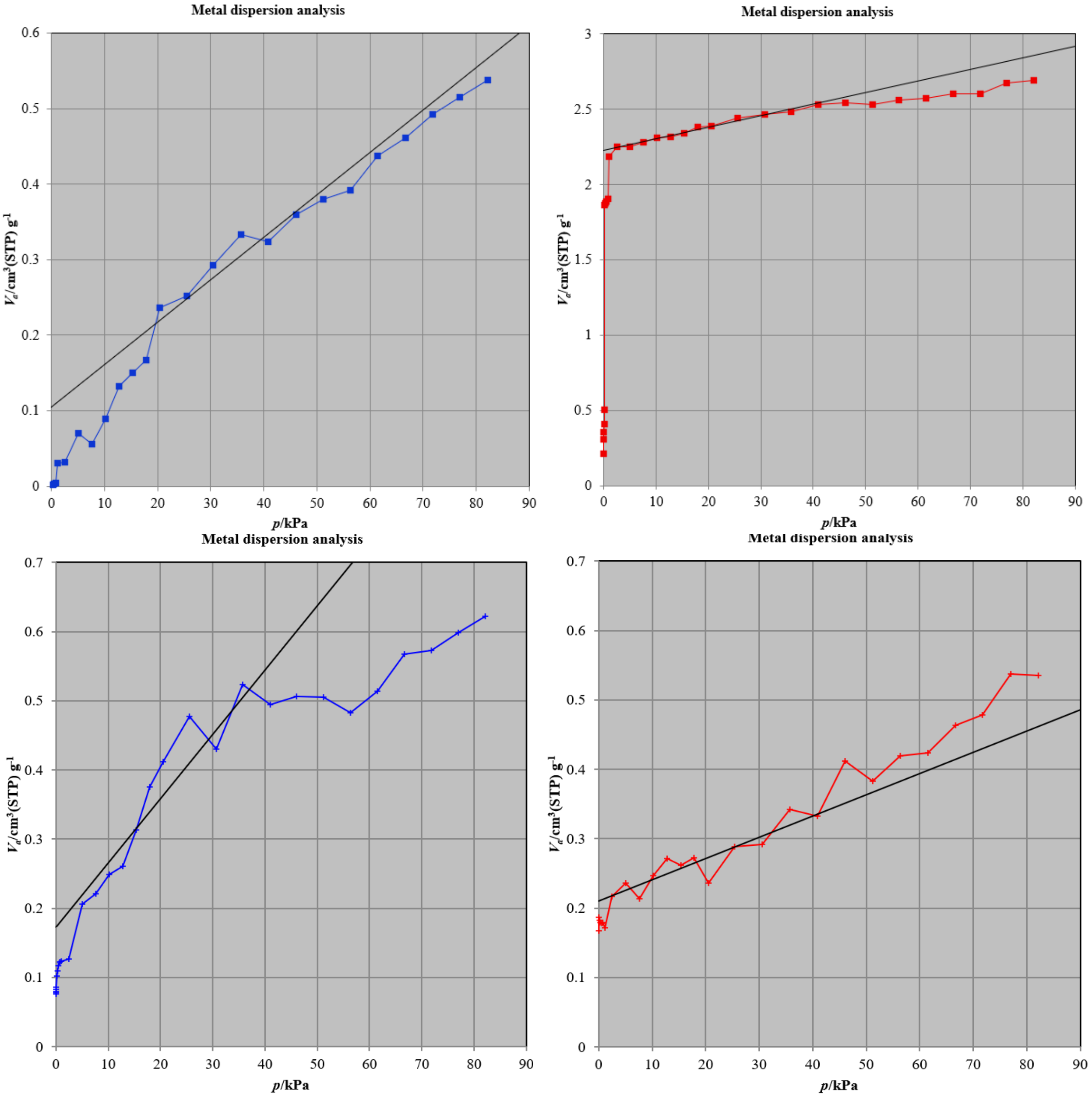

*Figure S15. H_2 Chemisorption of **Pt/C$_{600°C}$** (left top), **PtBi/C$_{600°C}$** (right top), **Pt/C$_{900°C}$** (left bottom) and **PtBi/C$_{900°C}$** (right bottom).*

Table S15. Summary of H_2 chemisorption data.

	Pt/C$_{600°C}$	PtBi/C$_{600°C}$	Pt/C$_{900°C}$	PtBi/C$_{900°C}$
Monolayer volume per mass of sample (cm^3/g)	0.1045	2.2261	0.1733	0.2103
Monolayer quantity per mass of sample (mmol/g)	0.0047	0.0994	0.0077	0.0094
Monolayer quantity per mass of Pt (mmol/g)	0.1545	2.9058	0.1441	0.2562

3.3.2. CO chemisorption

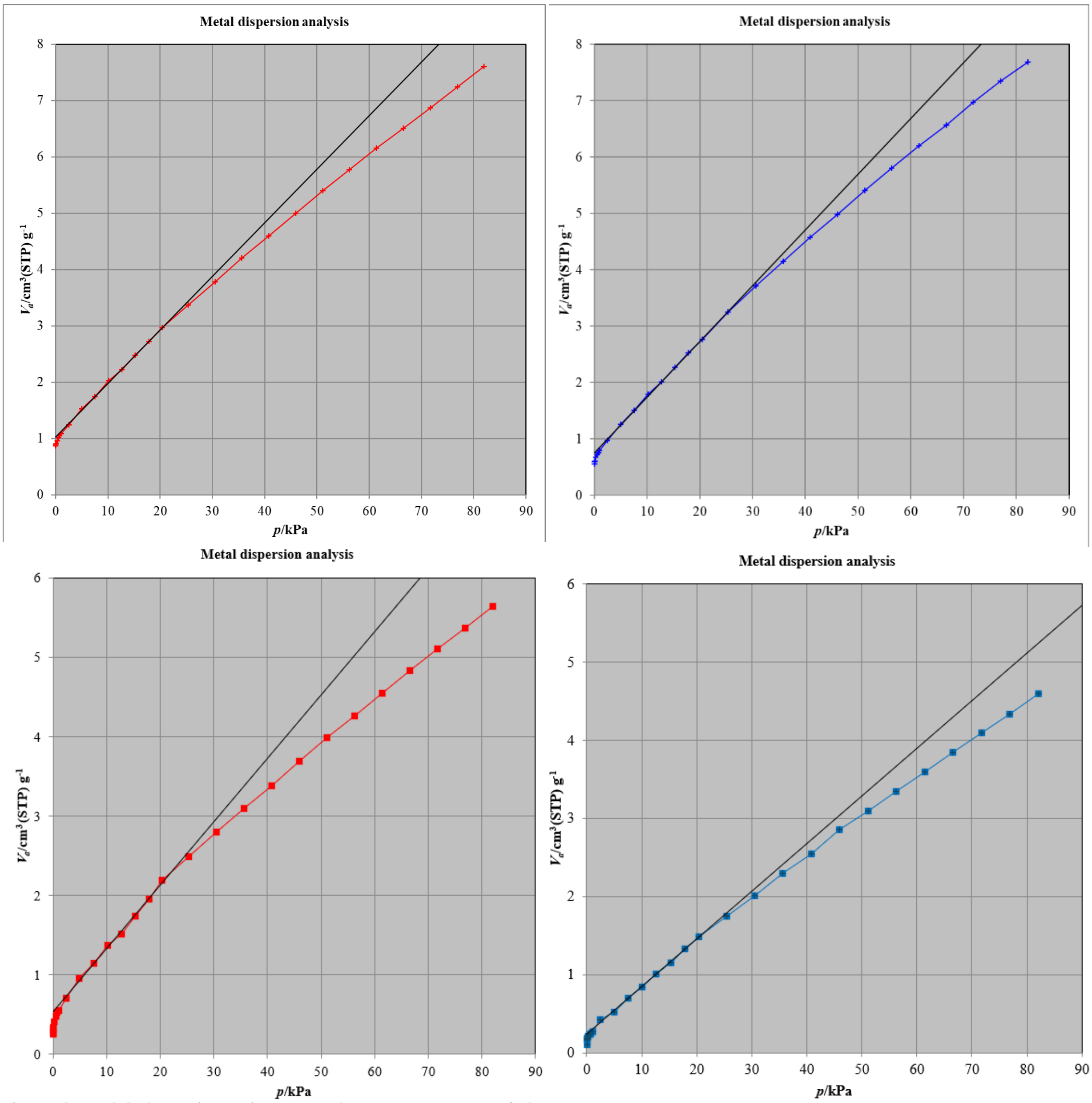

Figure S16. CO Chemisorption of ***Pt/C$_{600°C}$*** *(left) and* ***PtBi/C$_{900°C}$*** *(right).*

Table S16. Summary of CO chemisorption data.

	**Pt/C$_{600°C}$**	**PtBi/C$_{600°C}$**	**Pt/C$_{600°C}$** spent	**PtBi/C$_{600°C}$** spent
Monolayer volume per mass of sample (cm^3/g)	1.0294	0.7491	0.5372	0.2331
Monolayer quantity per mass of sample (mmol/g)	0.0459	0.0334	0.0240	0.0104
Monolayer quantity per mass of Pt (mmol/g)	1.5217	0.9778	0.7941	0.3043

3.4. CO-IR probe on SiO_2-supported materials

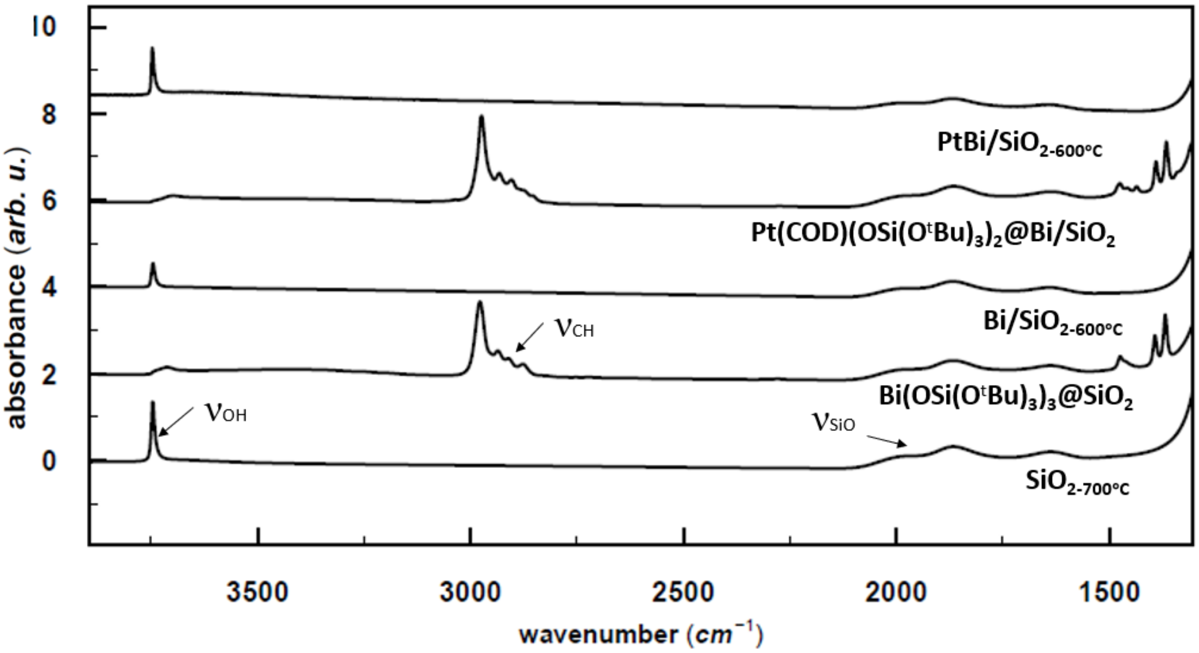

*Figure S17. FTIR spectrum of the pristine SiO_2 support, the grafted $Bi(OSi(OtBu)_3)_3$ before ($Bi(OSi(O^tBu)_3)_3@SiO_2$) and after (**$Bi/SiO_{2\text{-}600°C}$**) synthetic air treatment at elevated temperature, as well as [$Pt(COD)(OSi(O^tBu)_3)_2$] grafted on Bi/SiO_2 before ($Pt(COD)(OSi(O^tBu)_3)_2@Bi/SiO_2$) and after (**$PtBi/SiO_{2\text{-}600°C}$**) hydrogen treatment. The peaks around 3700 cm^{-1} are consistent with the presence of surface OH groups, which are consumed upon grafting and regenerated upon thermal treatments. The signals around 3000 cm^{-1} are assigned to the C-H stretching of the ^{t}Bu group, while the ones at 2000-1600 cm^{-1} originate from Si-O stretches.[6]*

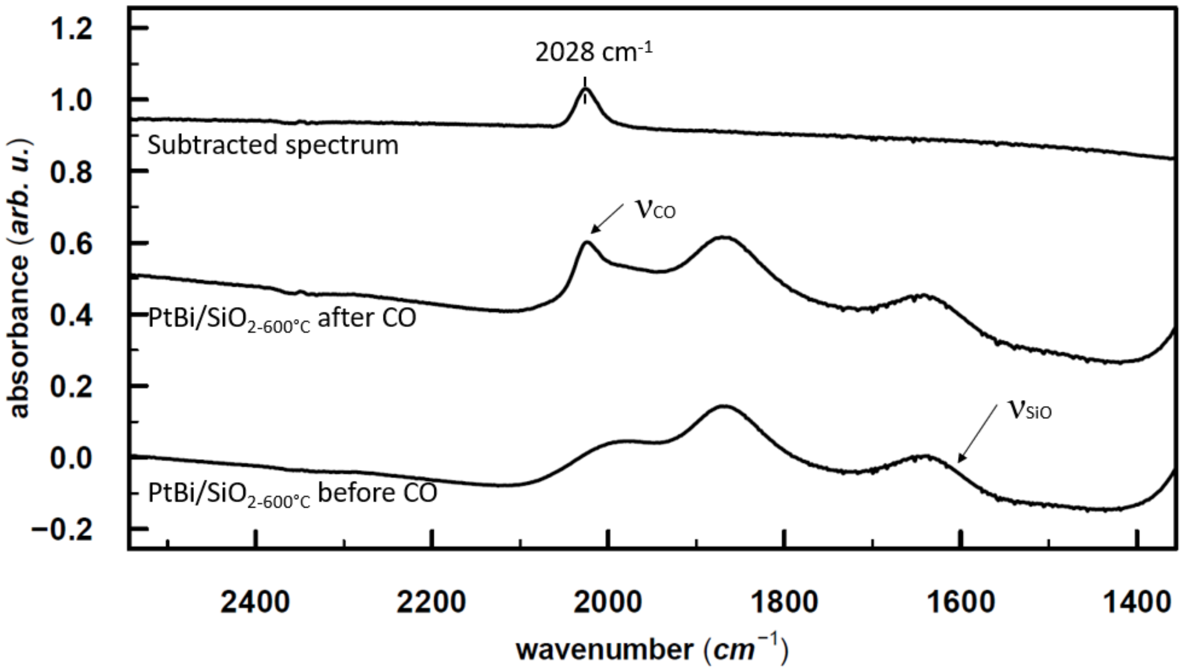

*Figure S18. FTIR spectrum in the 2500-1400 cm^{-1} region of **$PtBi/SiO_{2\text{-}600°C}$** before (bottom) and after (middle) exposure to 25 mbar of CO. While the appearance of the Si-O stretches is invariant, an additional signal at 2028 cm^{-1} is present after exposure to CO and clearly visible in the subtraction spectrum (top).*

3.5. X-Ray absorption spectroscopy

3.5.1. Pt L_3-edge

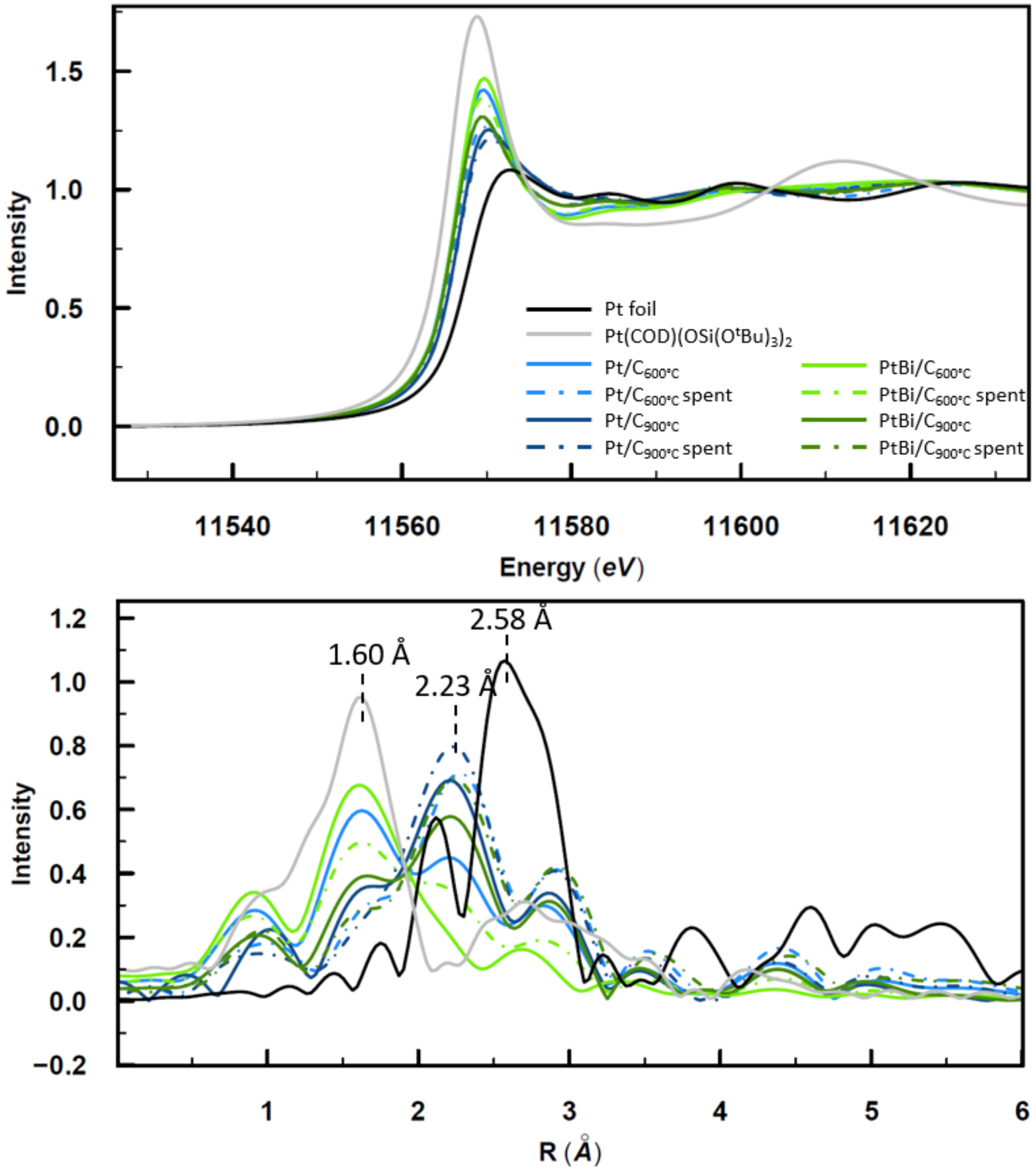

Figure S17. Top: Ex situ Pt L_3-edge XANES spectra of all prepared ***Pt/C*** *catalysts, Pt foil and Pt(COD)(OSi(O^{t}Bu)$_3$)$_2$ references; and their radial distance R (Å) (bottom).*

Table S17. Fitting parameters of Pt L_3-edge XANES data.

Material	% $Pt(II)(COD)(OSi(O^tBu)_3)_2$	% Pt(0) foil	R factor
**Pt/C$_{600°C}$**	58%	42%	0.00190
**Pt/C$_{600°C}$** spent	37%	62%	0.00184
**Pt/C$_{900°C}$**	30%	70%	0.00211
**Pt/C$_{900°C}$** spent	25%	75%	0.00244
**PtBi/C$_{600°C}$**	67%	33%	0.00221
**PtBi/C$_{600°C}$** spent	54%	46%	0.00265
**PtBi/C$_{900°C}$**	41%	59%	0.00168
**PtBi/C$_{900°C}$** spent	38%	62%	0.00182

3.5.2. Bi L_3-edge

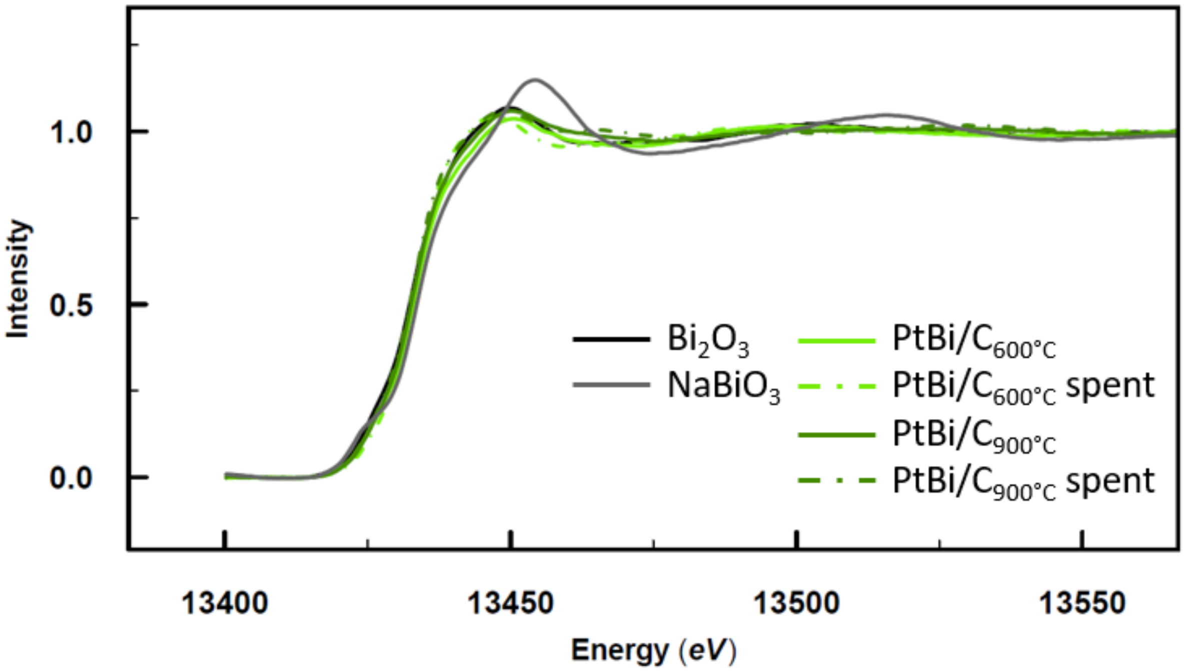

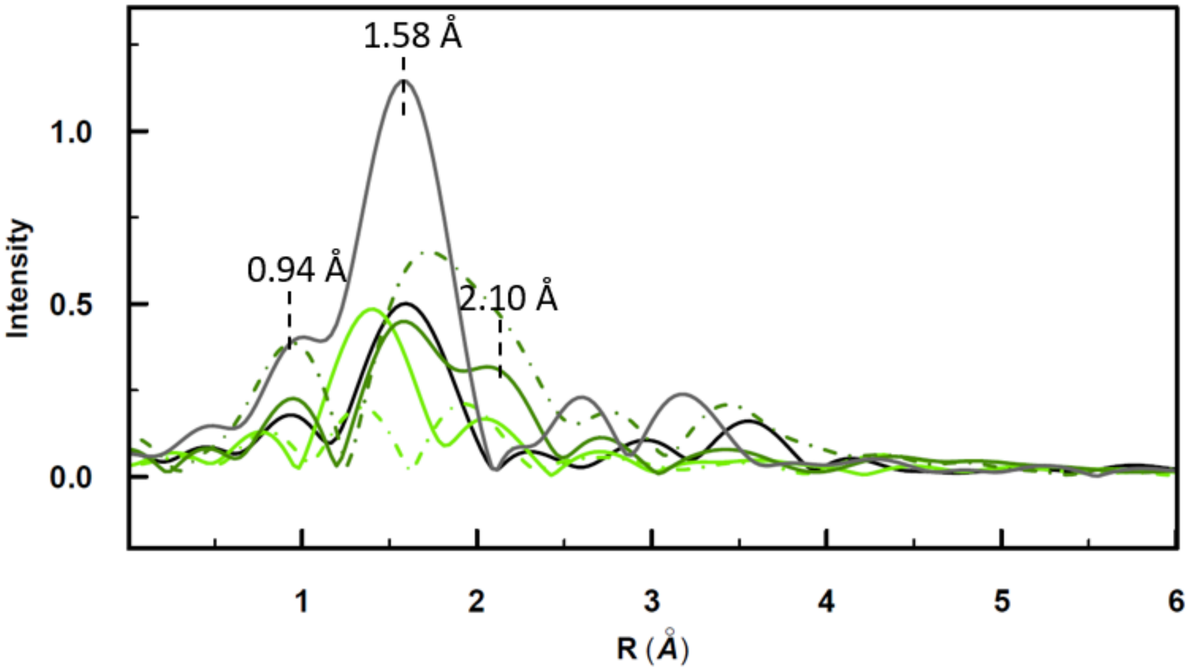

Figure S18. Top: Ex situ Bi L_3-edge XANES spectra of all prepared ***PtBi/C*** *catalysts, Bi_2O_3 and $NaBiO_3$ references; and their radial distance R (Å) (bottom).*

3.5.3. Bi L_2-edge

3.5.4.

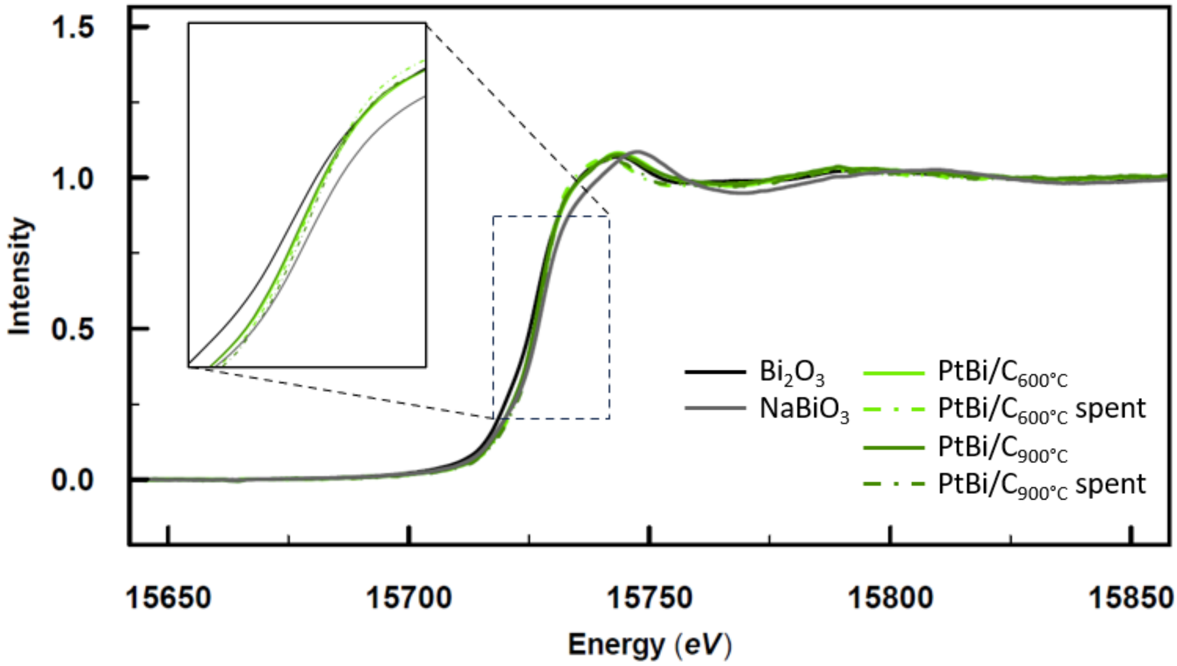

*Figure S19. Top: Ex situ Bi L_2-edge XANES spectra of all prepared **PtBi/C** catalysts, Bi_2O_3 and $NaBiO_3$ references.*

Table S18 Fitting of Bi L_2-edge XANES data.

Material	% Bi (III)	% Bi(V)	R factor
**PtBi/C$_{600°C}$**	87%	13%	0.00267
**PtBi/C$_{600°C}$** spent	100%		0.00343
**PtBi/C$_{900°C}$**	96%	4%	0.00098
**PtBi/C$_{900°C}$** spent	100%		0.00093

4. Catalytic tests

4.1. Experimental procedure

Oxidation of 2-methoxyethanol

A pressure-resistant glass tube was loaded with around 30 mg of catalyst, 0.252 mL of 2-methoxyethanol, and 0.812 mL of distilled water (30 wt% substrate in water, 1 : 200 Pt : substrate ratio). The reaction was then carried out at 50 °C, 6 bar of synthetic air, and a stirring rate of 1000 rpm for 9 h. The solution was then cooled to RT, 0.812 mL of distilled water were added, and the mixture was filtered. The solution was then analyzed by ^{1}H NMR in a 90 % H_2O, 10% D_2O mixture: 0.400 mL of the reaction mixture, 0.050 mL of D_2O, and 0.010 mL of acetone as standard.

Oxidation of prenol

A pressure-resistant glass tube was loaded with around 15 mg of catalyst, 0.133 mL of prenol, and 2.547 mL of distilled water (5 wt% substrate in water, 1 : 200 Pt : substrate ratio). The reaction was then carried out at 50 °C, 6 bar of synthetic air, and a stirring rate of 1000 rpm for 9 h. The solution was then cooled to RT, and the mixture was filtered. For the recycling tests, the catalyst was washed with distilled water, dried, and tested under the same conditions. The solution was then analyzed by ^{1}H NMR in a 90 % H_2O, 10% D_20 mixture: 0.400 mL of the reaction mixture, 0.050 mL of D_2O, and 0.010 mL of acetone as standard.

4.2. Catalytic results

Oxidation of 2-methoxyethanol

Table S19. Summary of catalytic test of 2-methoxyethanol.

Material	Conversion (%)	Selectivity to 2-methoxacetic acid
**Bi/C$_{600°C}$**	0	0
**Pt/C$_{600°C}$**	5.2	91.0
**Pt/C$_{750°C}$**	34.3	91.8
**Pt/C$_{900°C}$**	48.3	93.6
**PtBi/C$_{600°C}$**	22.8	95.5
**PtBi/C$_{750°C}$**	54.1	95.6
**PtBi/C$_{900°C}$**	58.3	98.6

Oxidation of prenol

Table S20. Summary of catalytic test prenol.

Material	Conversion (%)	Selectivity to prenal
**Pt/C$_{900°C}$** 1st cycle	80.8	98.5
**Pt/C$_{900°C}$** 2nd cycle	89.5	85.3
**Pt/C$_{900°C}$** 3rd cycle	89.9	83.7
**Pt/C$_{900°C}$** 4th cycle	87.0	81.2
**Pt/C$_{900°C}$** 5th cycle	86.0	85.6
**PtBi/C$_{600°C}$**	61.0	95.3
**PtBi/C$_{900°C}$** (95 V% prenol in H_2O)	7.4	100
**PtBi/C$_{900°C}$** 1st cycle	93.1	98.3
**PtBi/C$_{900°C}$** 2nd cycle	93.0	97.4
**PtBi/C$_{900°C}$** 3rd cycle	91.2	98.1
**PtBi/C$_{900°C}$** 4th cycle	93.1	99.1
**PtBi/C$_{900°C}$** 5th cycle	87.7	99.0

4.3. ^{1}H NMR of catalytic tests

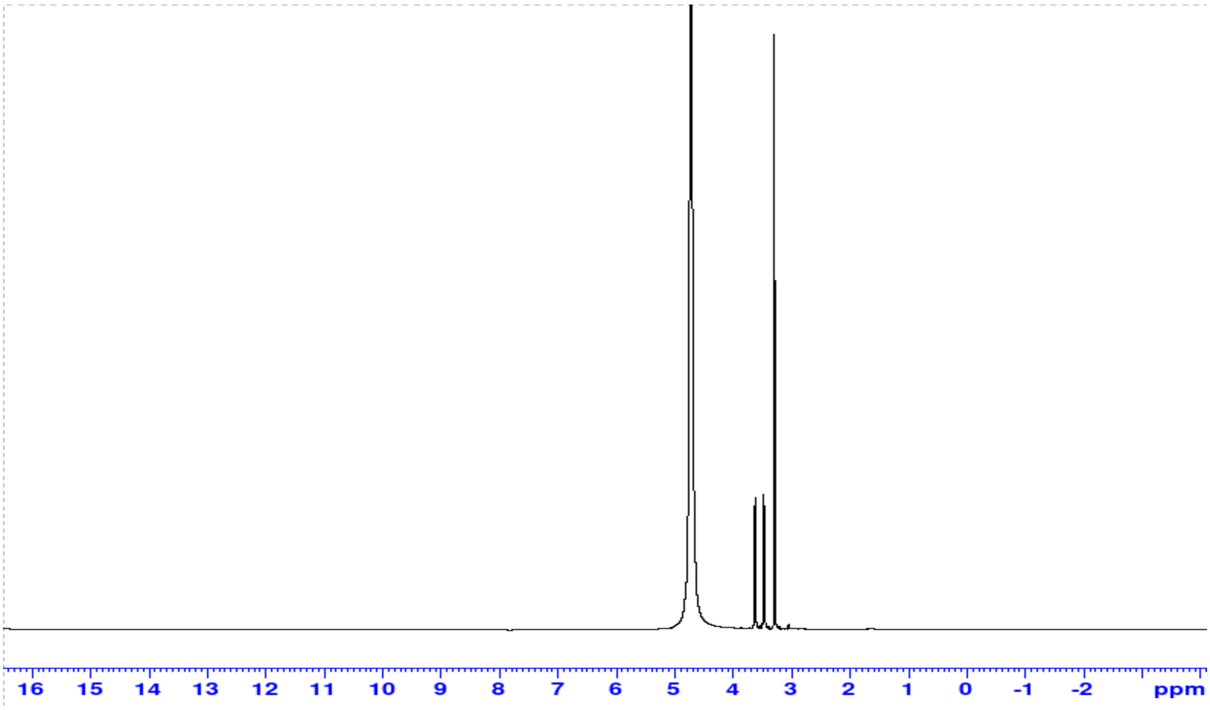

Figure S20. ^{1}H NMR spectrum of catalytic results from 2-methoxy ethanol oxidation with ***Bi/C$_{600°C}$****.*

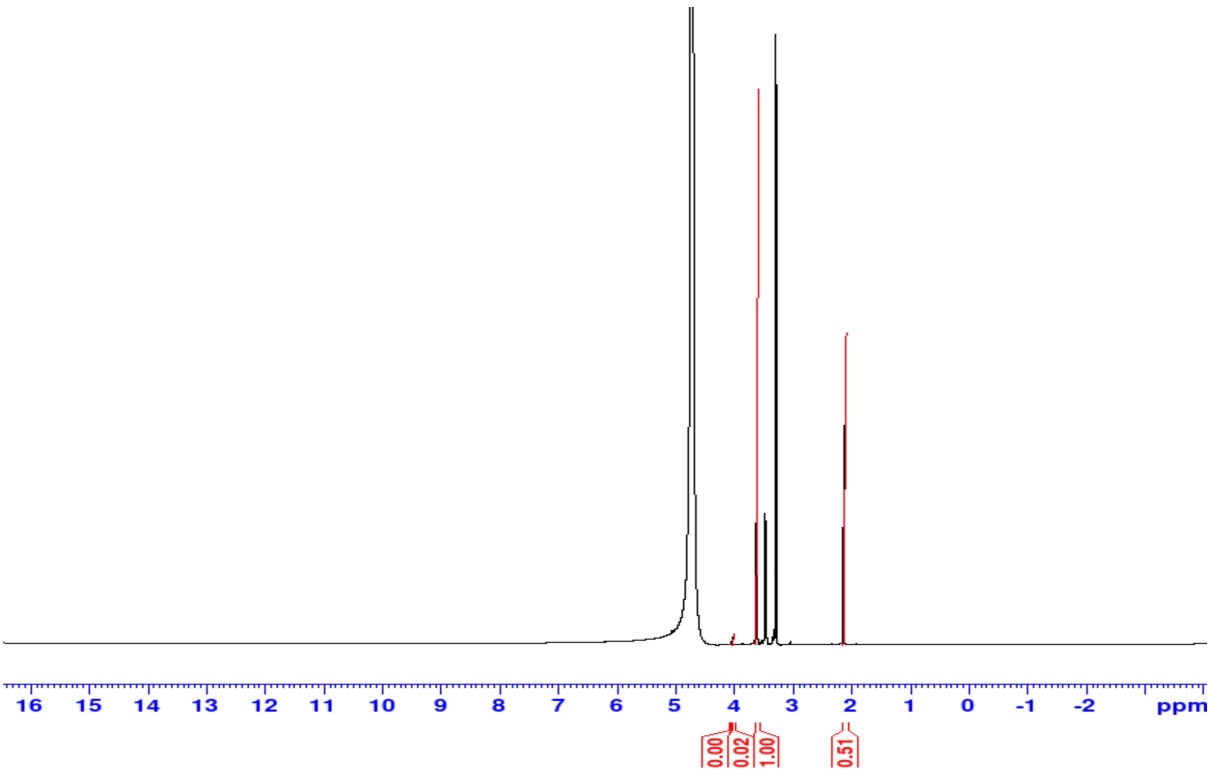

Figure S21. ^{1}H NMR spectrum of catalytic results from2-methoxy ethanol oxidation with ***Pt/C$_{600°C}$****.*

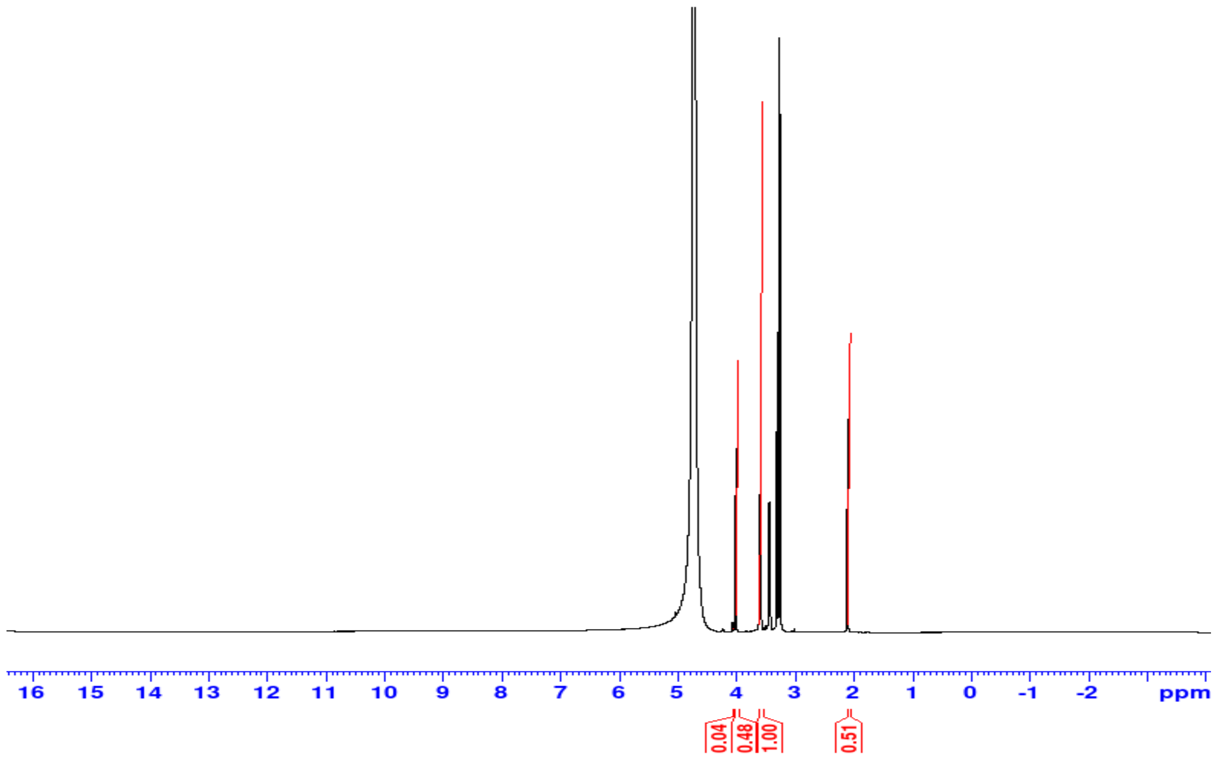

Figure S22. ^{1}H *NMR spectrum of catalytic results from 2-methoxy ethanol oxidation with* ***Pt/C$_{750°C}$***.

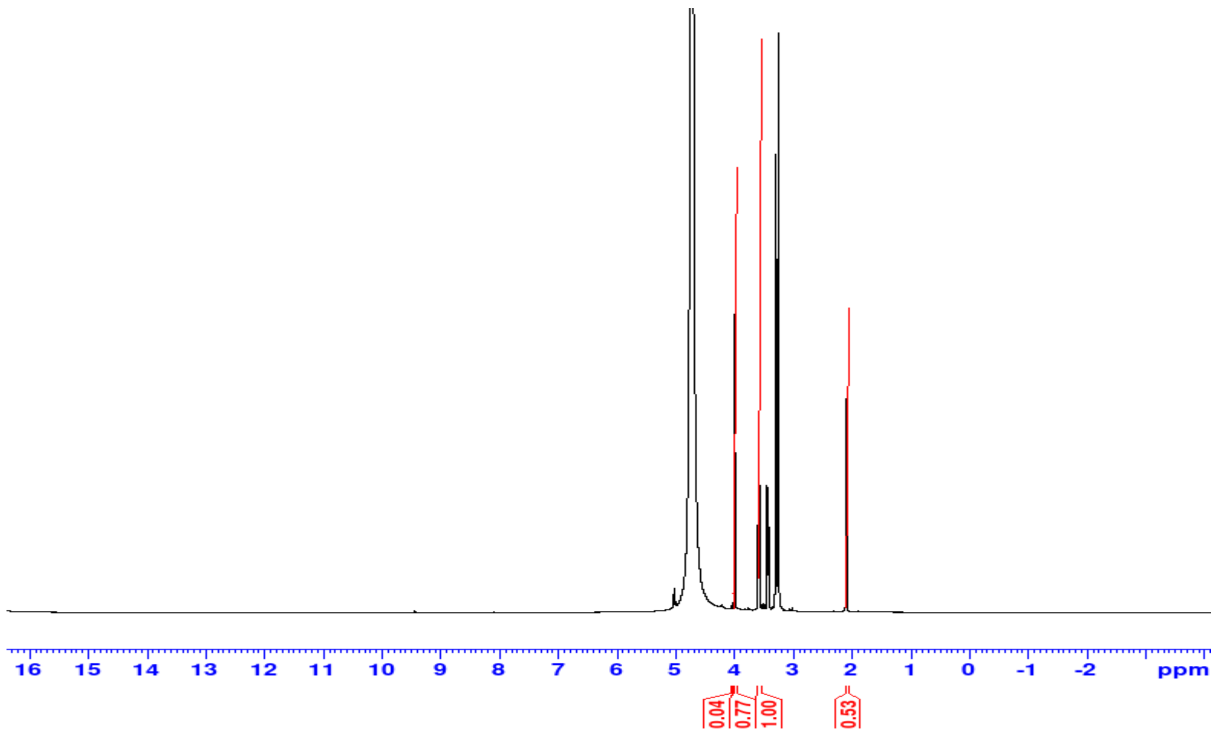

Figure S23. ^{1}H *NMR spectrum of catalytic results from 2-methoxy ethanol oxidation with* ***Pt/C$_{900°C}$***.

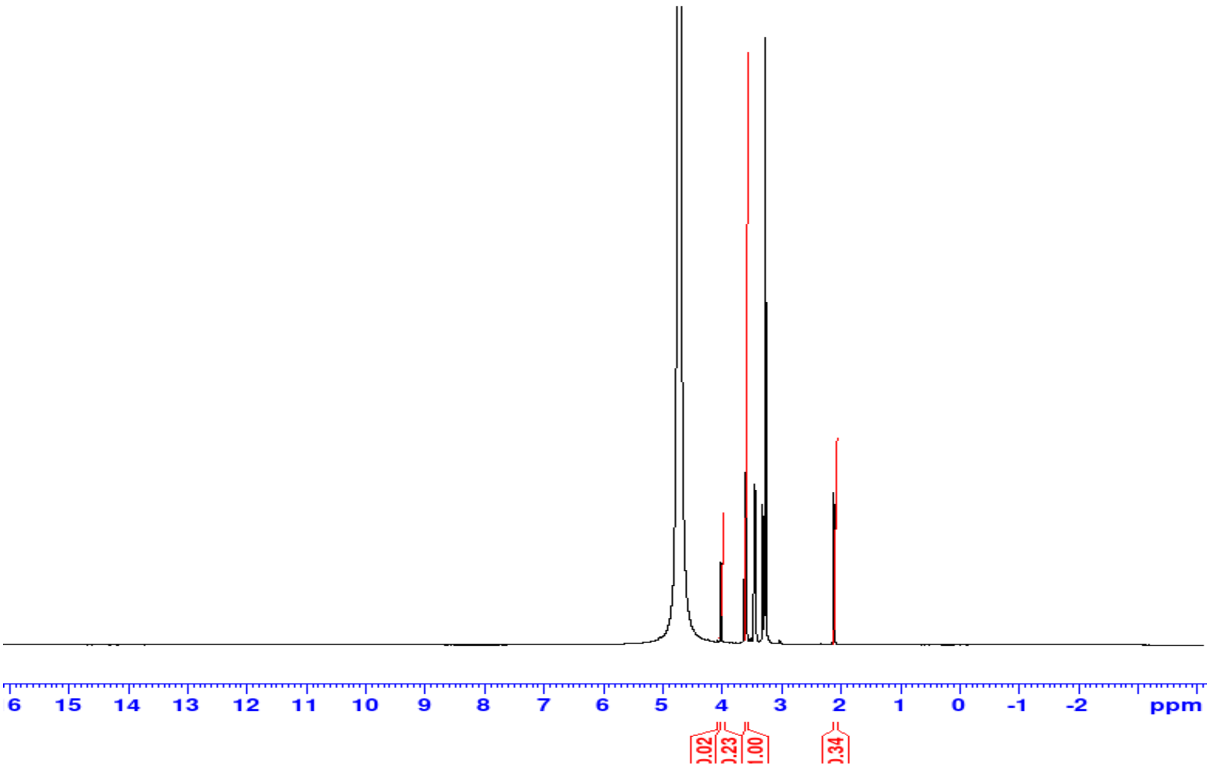

Figure S24. [1]*H NMR spectrum of catalytic results from 2-methoxy ethanol oxidation with* ***PtBi/C**$_{600°C}$*.

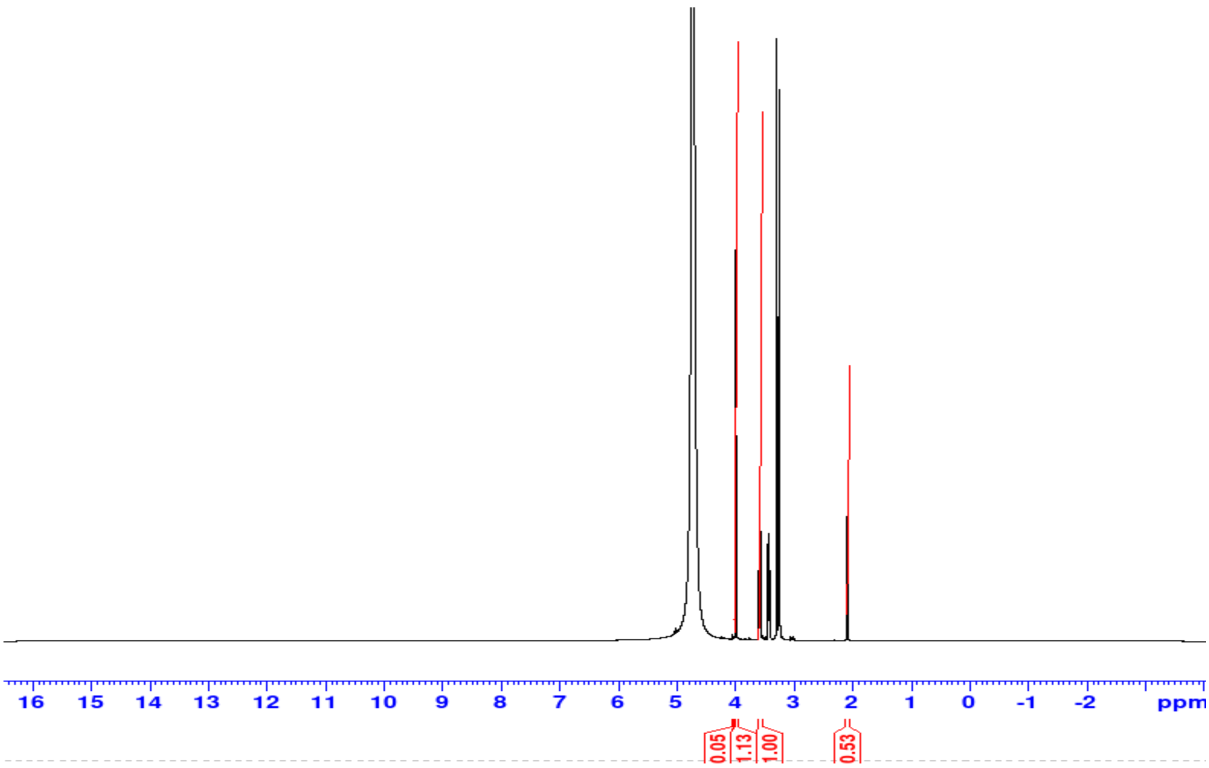

Figure S25. [1]*H NMR spectrum of catalytic results from 2-methoxy ethanol oxidation with* ***PtBi/C**$_{750°C}$*.

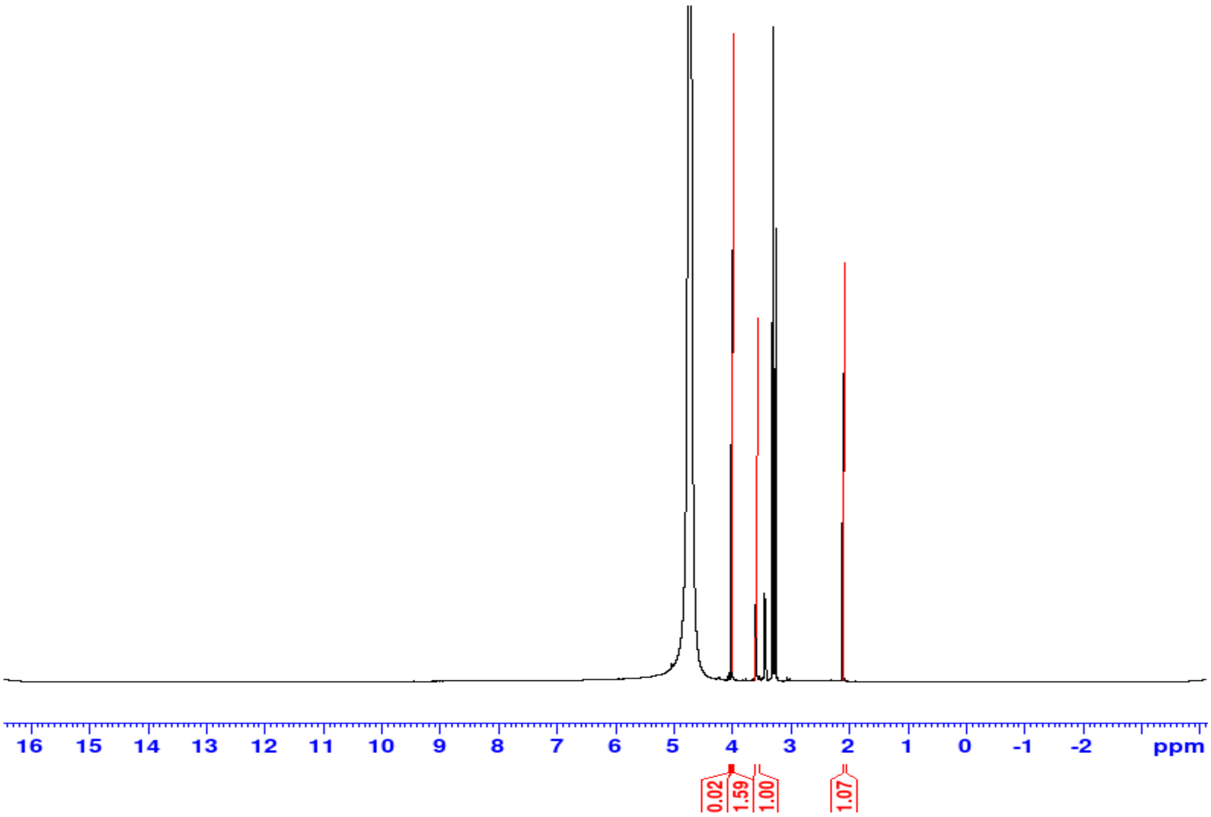

Figure S26. [1]*H NMR spectrum of catalytic results from 2-methoxy ethanol oxidation with* ***PtBi/C***$_{900°C}$.

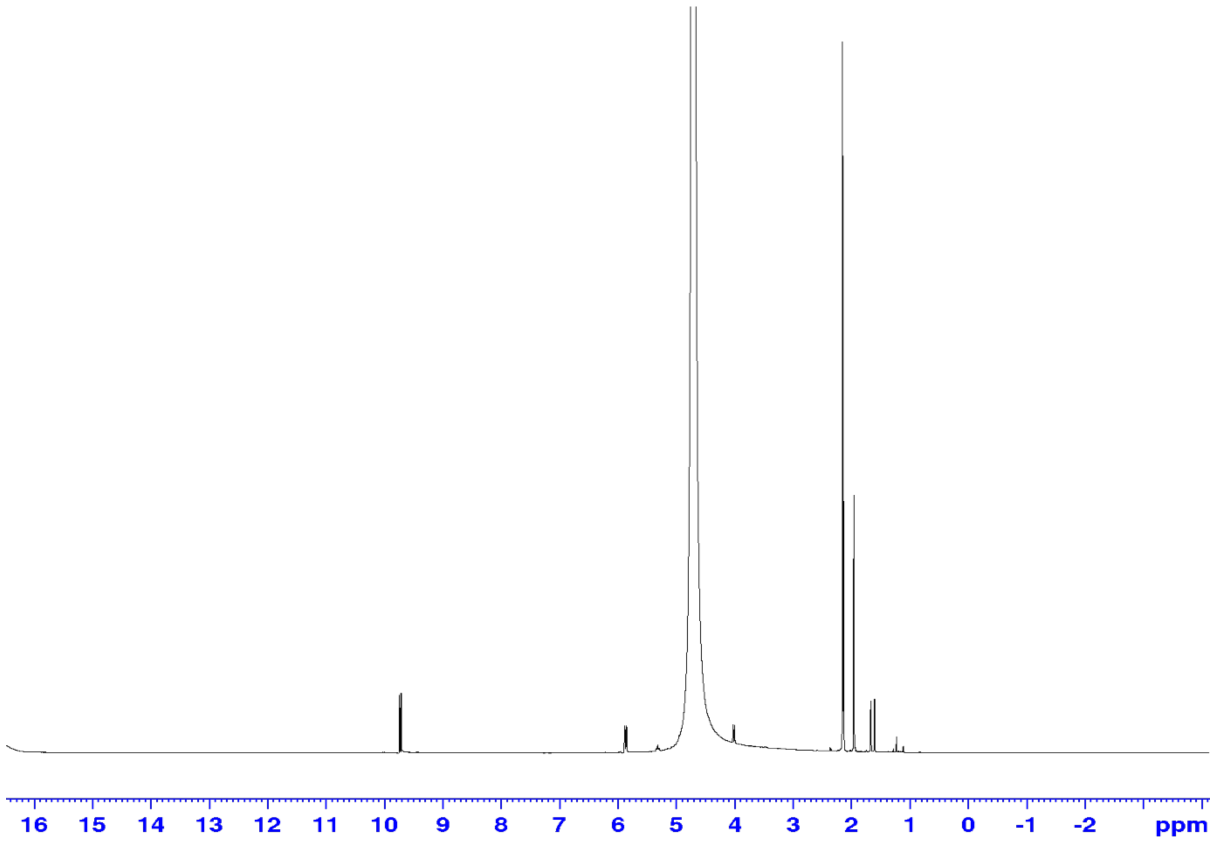

Figure S29 ^{1}H NMR spectrum of catalytic results from the first cycle of prenol oxidation with ***Pt/C$_{900°C}$****.*

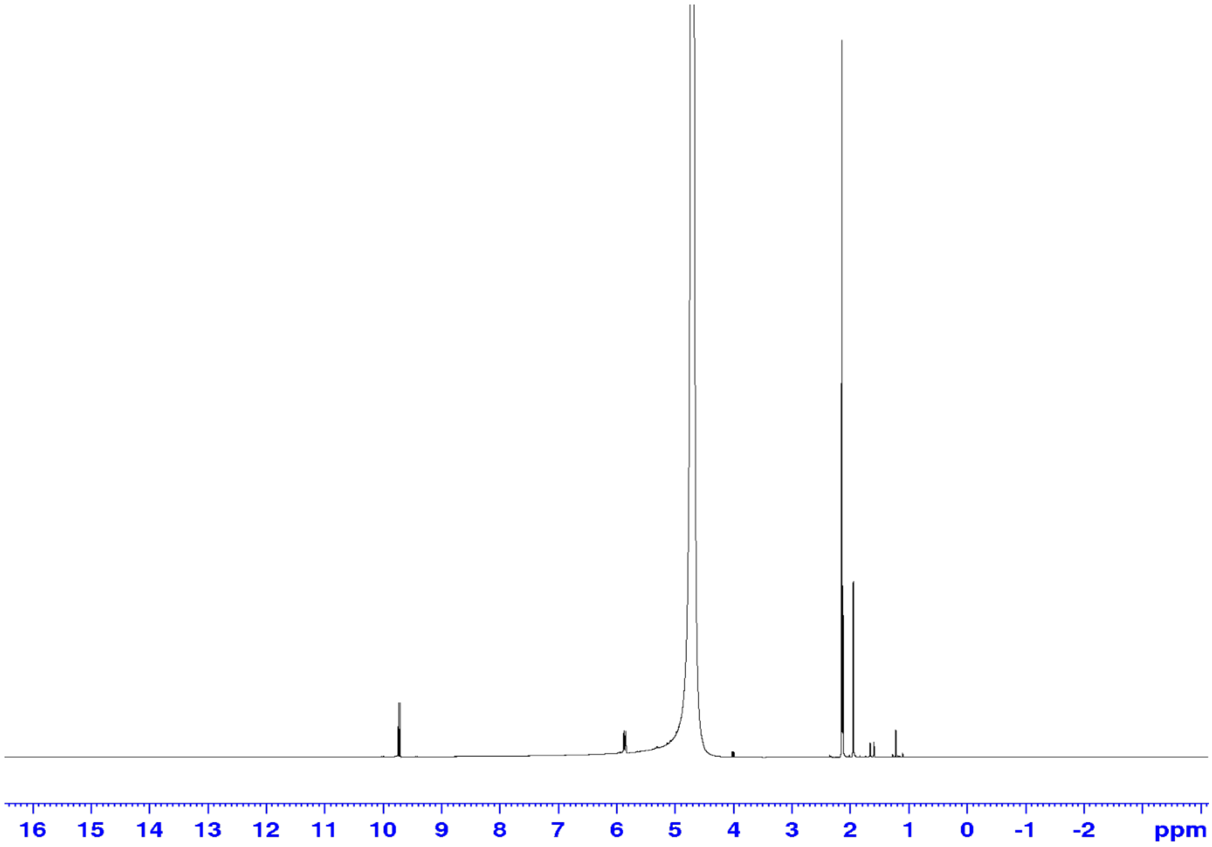

Figure S30. ^{1}H NMR spectrum of catalytic results from the second cycle of prenol oxidation with ***Pt/C$_{900°C}$****.*

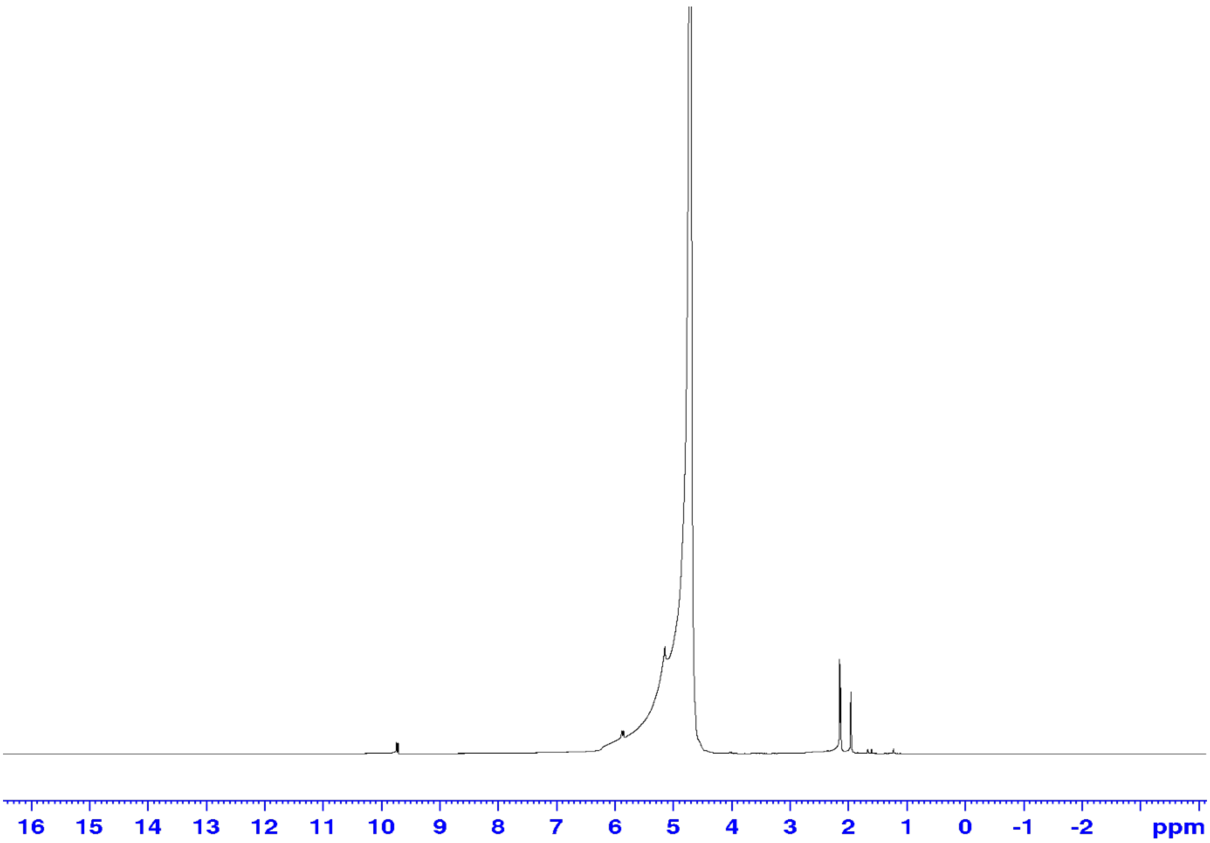

Figure S31. *^{1}H NMR spectrum of catalytic results from the third cycle of prenol oxidation with* ***Pt/C$_{900°C}$***.

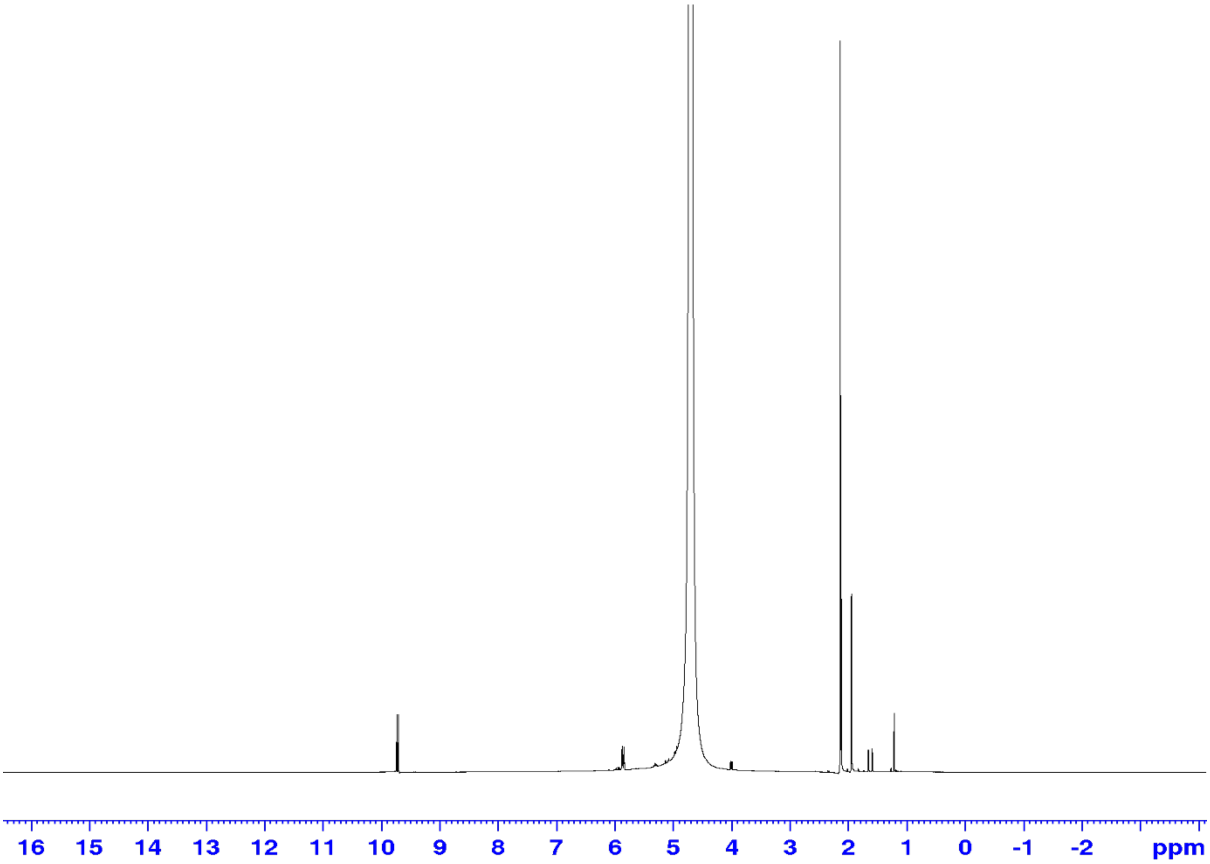

Figure S32. *^{1}H NMR spectrum of catalytic results from the fourth cycle of prenol oxidation with* ***Pt/C$_{900°C}$***.

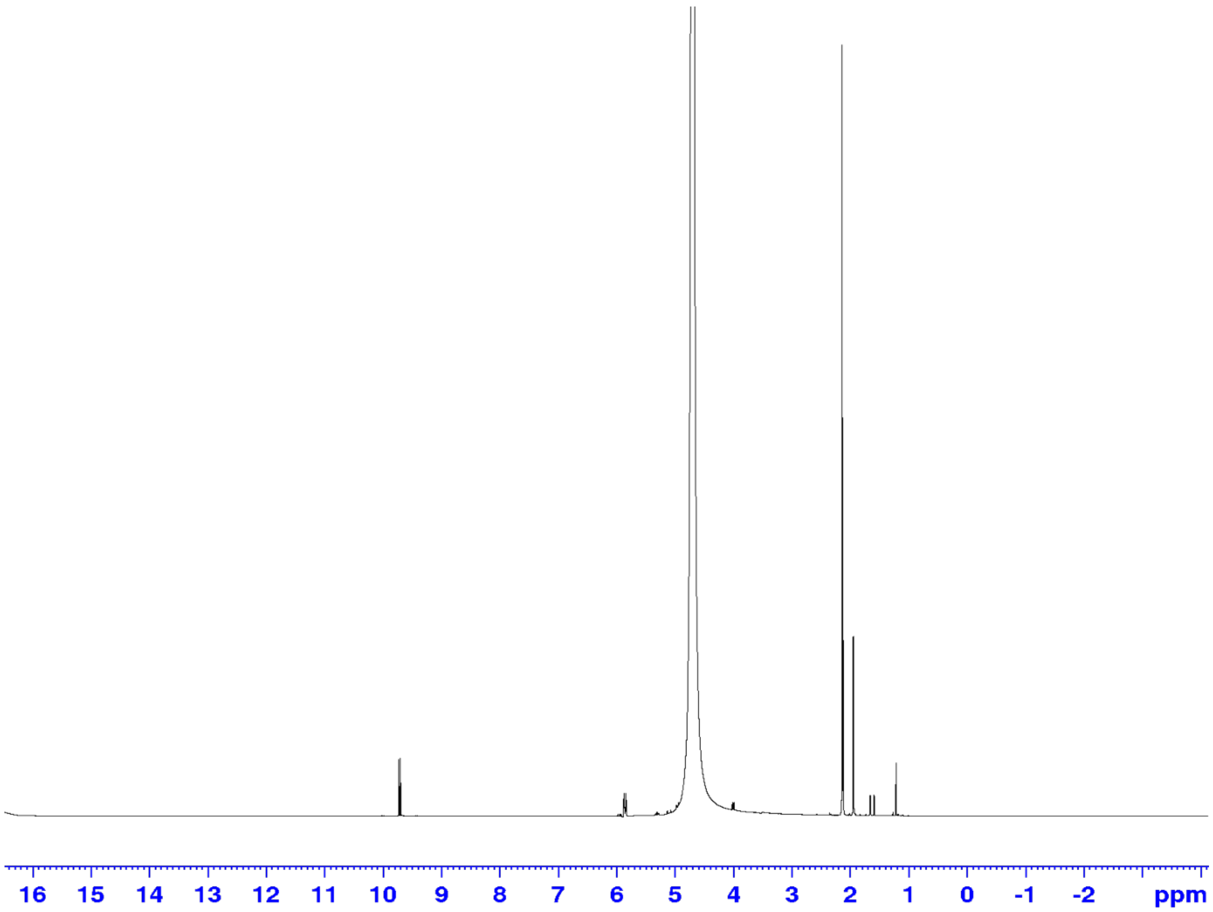

Figure S33. ^{1}H NMR spectrum of catalytic results from the fifth cycle of prenol oxidation with ***Pt/C$_{900°C}$***.

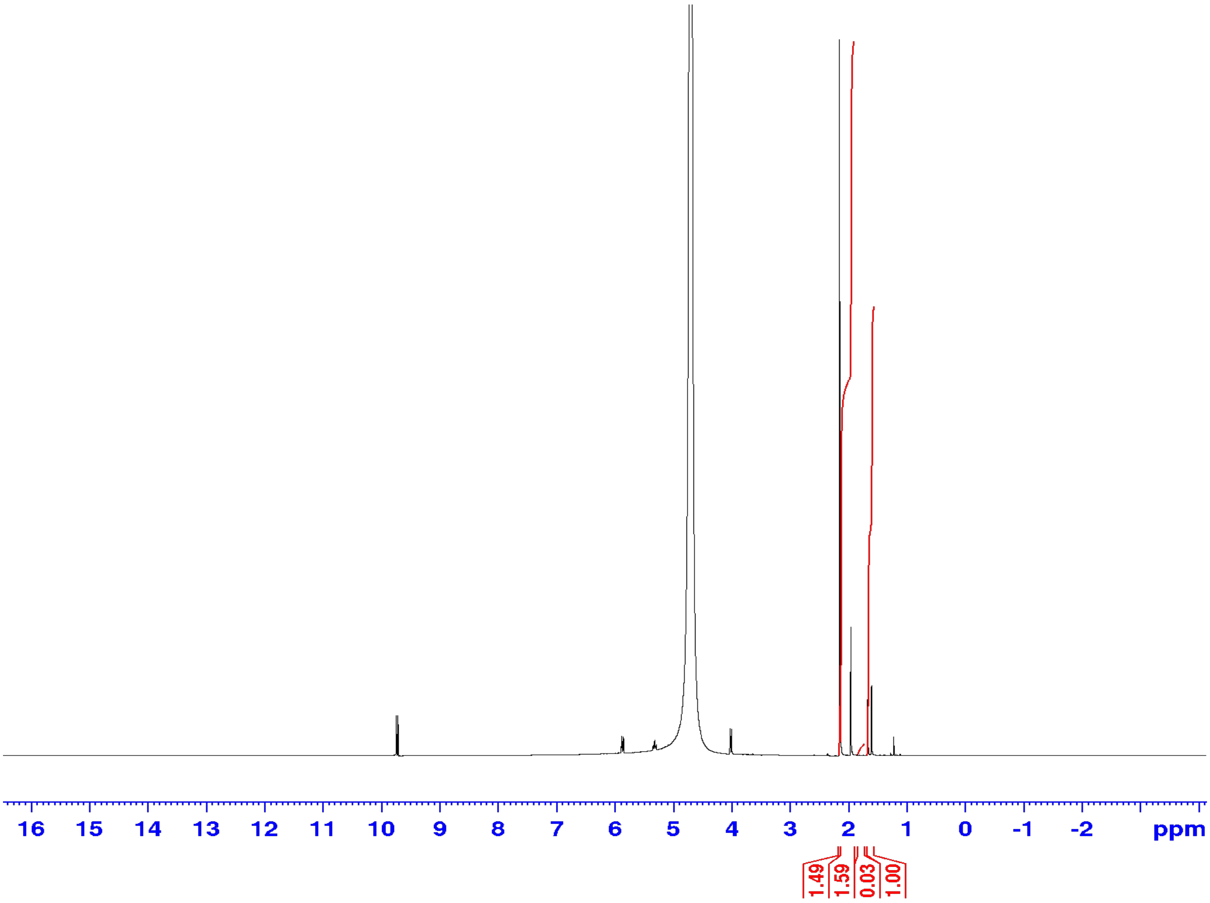

*Figure S34. ^{1}H NMR spectrum of catalytic results from prenol oxidation with **PtBi/C600°C**.*

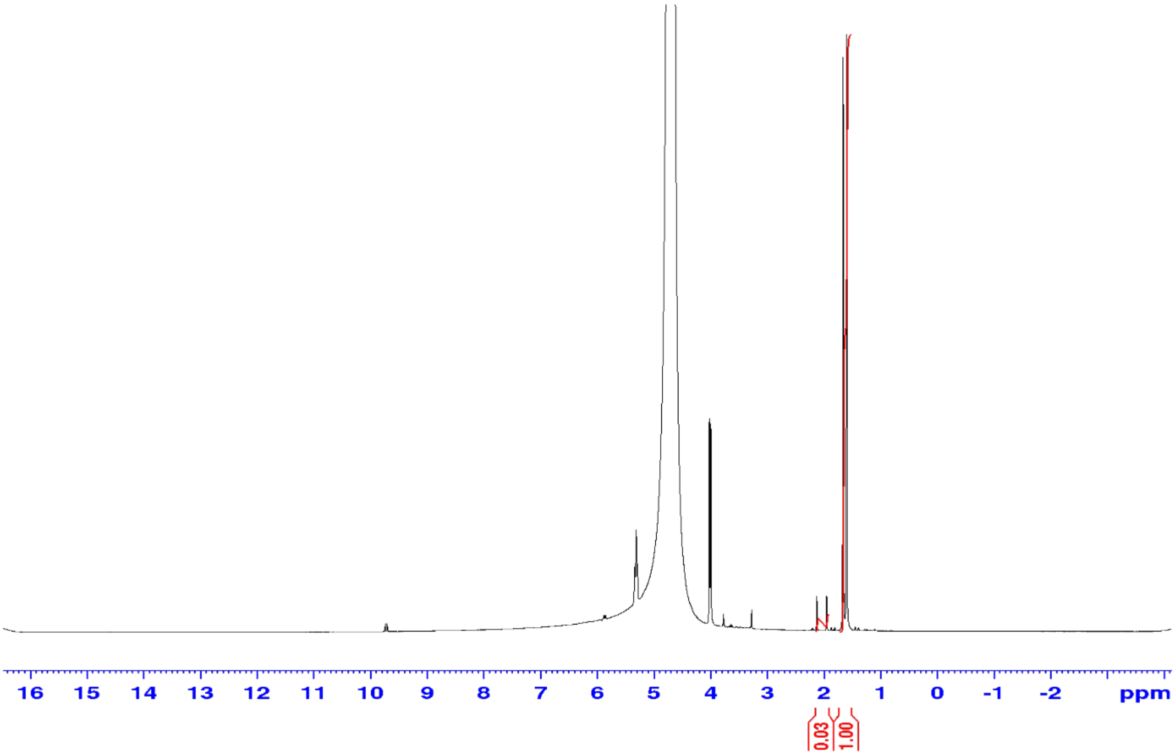

*Figure S35 ^{1}H NMR spectrum of catalytic results from the prenol oxidation with **PtBi/C900°C**. using 95 V% of prenol in water.*

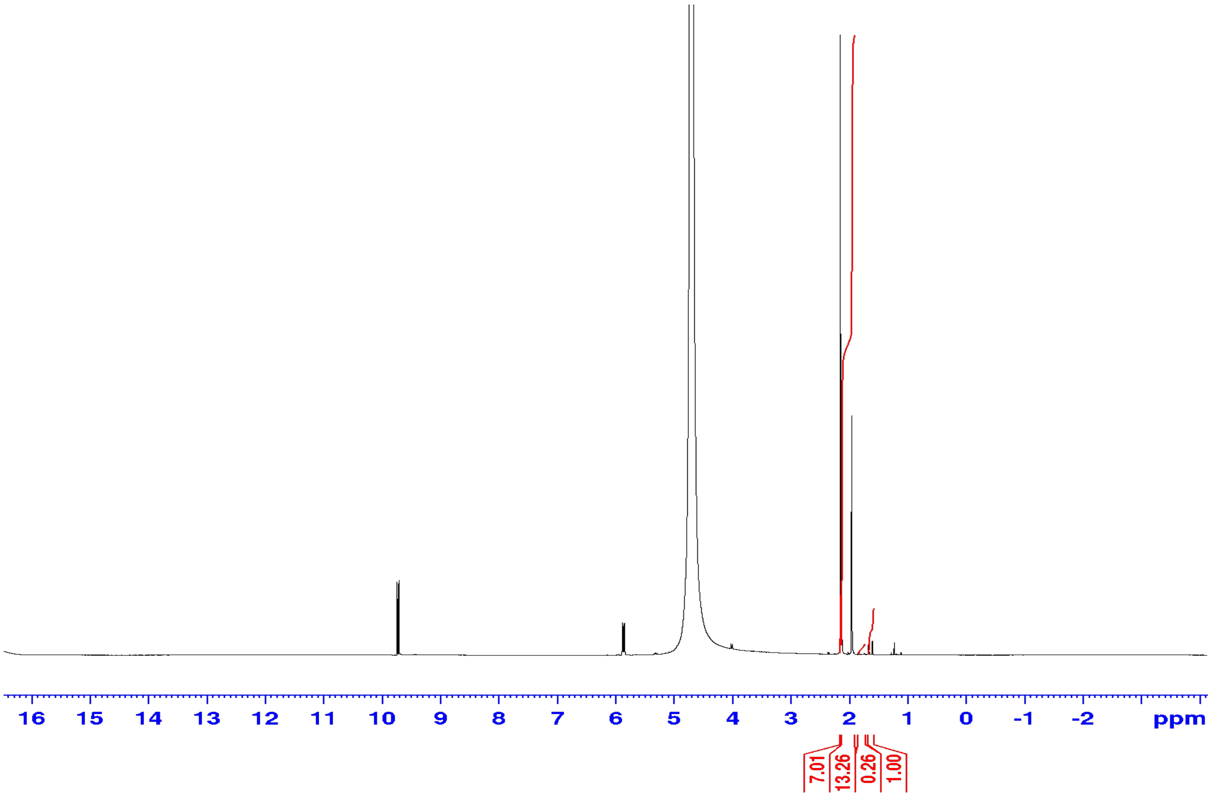

Figure S36. [1]*H NMR spectrum of catalytic results from the first cycle of prenol oxidation with* ***PtBi/C***$_{\mathbf{900°C}}$.

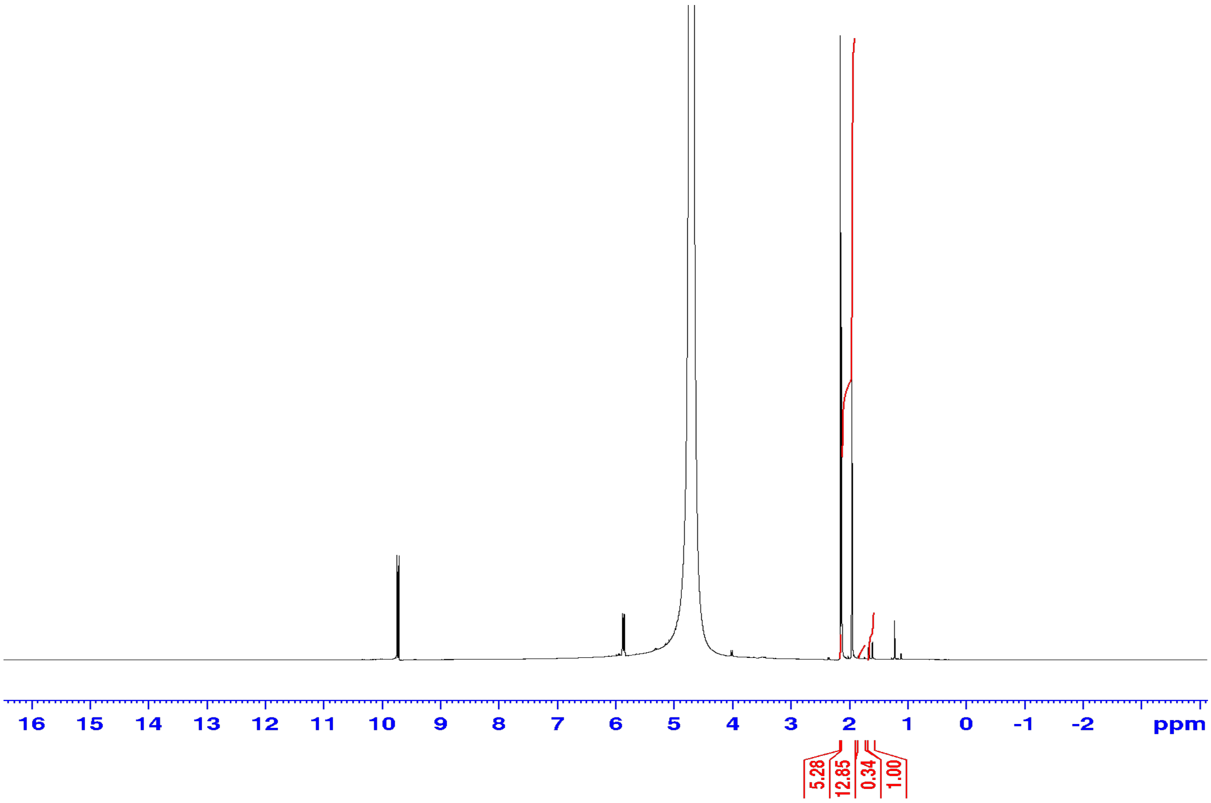

Figure S37. [1]*H NMR spectrum of catalytic results from the second cycle of prenol oxidation with* ***PtBi/C***$_{\mathbf{900°C}}$.

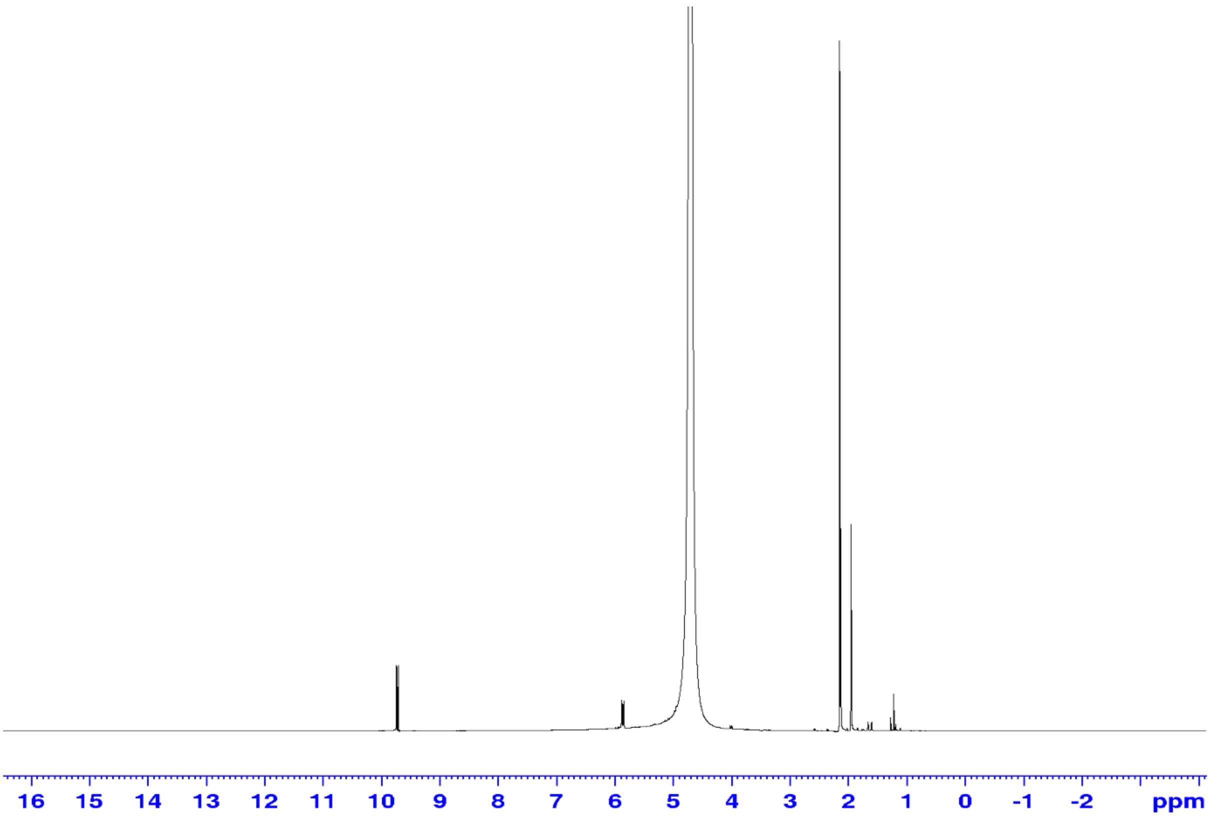

Figure S38. ^{1}H NMR spectrum of catalytic results from the third cycle of prenol oxidation with ***PtBi/C$_{900°C}$***.

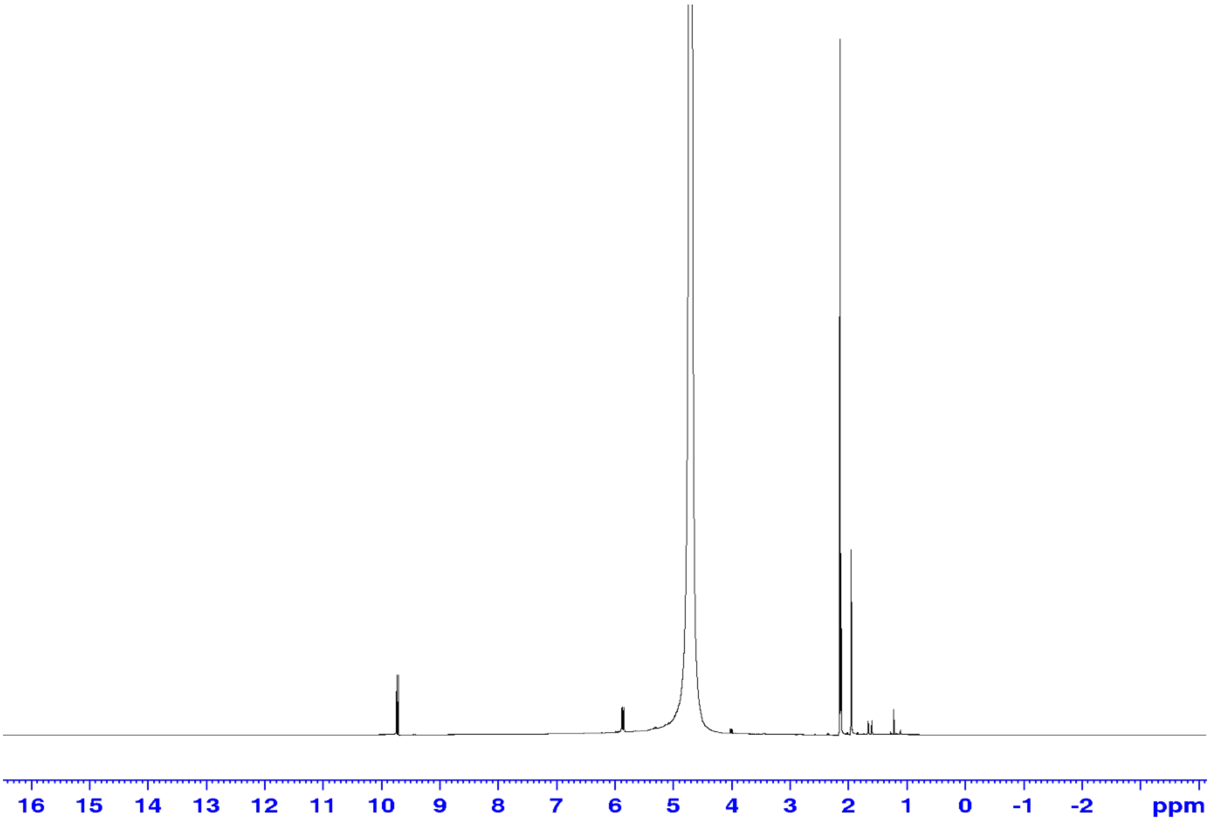

Figure S39. ^{1}H NMR spectrum of catalytic results from the fourth cycle of prenol oxidation with ***PtBi/C$_{900°C}$***.

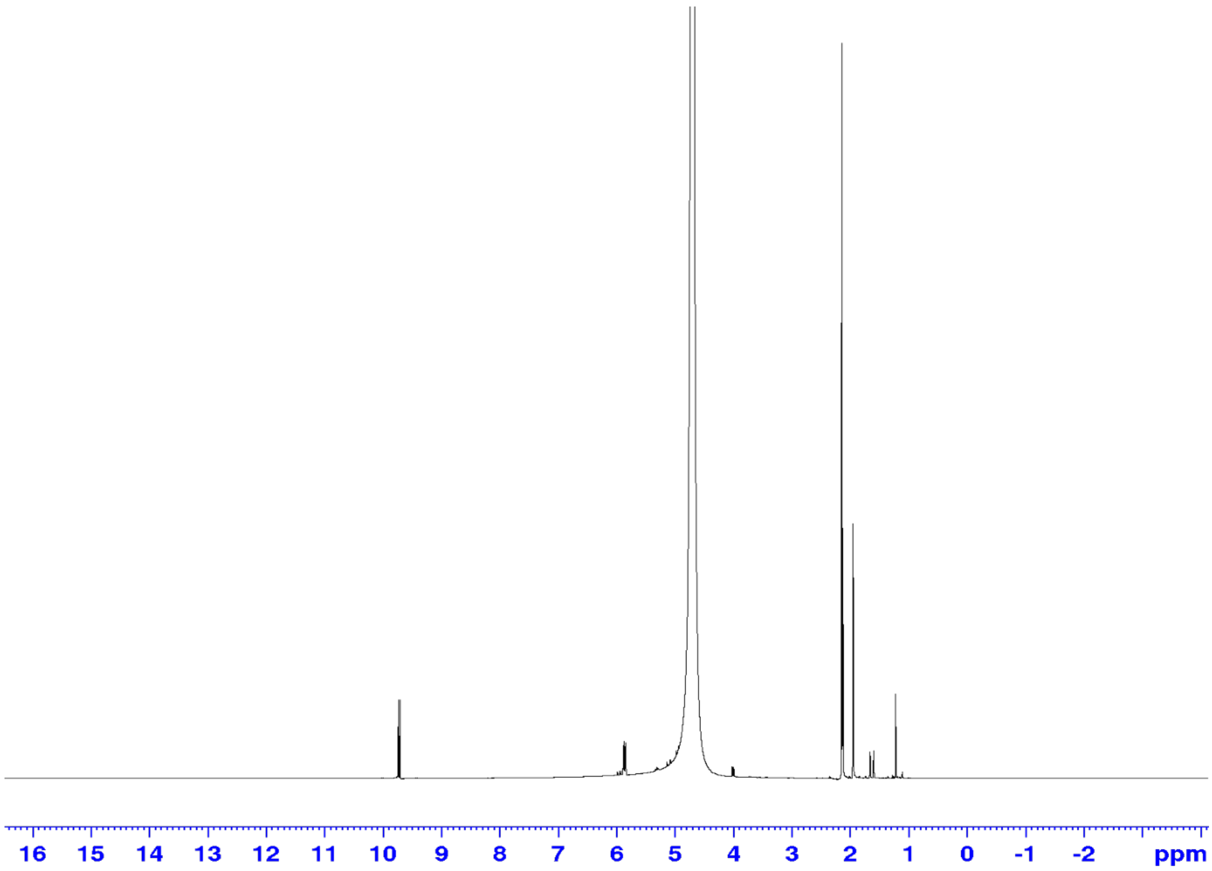

Figure S40. ^{1}H NMR spectrum of catalytic results from the fifth cycle of prenol oxidation with ***PtBi/C$_{900°C}$***.

[1] K. W. Terry, K. Su, T. Don Tilley, A. L. Rheingold, *Polyhedron* **1998**, *17*, 891-897.
[2] D. A. Ruddy, J. Jarupatrakorn, R. M. Rioux, J. T. Miller, M. J. McMurdo, J. L. McBee, K. A. Tupper, T. D. Tilley, *Chemistry of Materials* **2008**, *20*, 6517-6527.
[3] A. K. McMullen, T. D. Tilley, A. L. Rheingold, S. J. Geib, *Inorganic Chemistry* **1989**, *28*, 3772-3774.
[4] J. J. Sandoval, P. Palma, E. Álvarez, J. Cámpora, A. Rodríguez-Delgado, American Chemical Society, **2016**.
[5] H. Zhang, Y. Li, Y. Zhao, G. Li, F. Zhang, *ACS Applied Materials & Interfaces* **2019**, *11*, 27846-27853.
[6] L. Rochlitz, K. Searles, J. Alfke, D. Zemlyanov, O. V. Safonova, C. Copéret, *Chemical Science* **2020**, *11*, 1549-1555.
